# Structure–Activity Relationship Studies Based on Quinazoline Derivatives as EGFR Kinase Inhibitors (2017–Present)

**DOI:** 10.3390/ph16040534

**Published:** 2023-04-03

**Authors:** Alexandru Șandor, Ioana Ionuț, Gabriel Marc, Ilioara Oniga, Dan Eniu, Ovidiu Oniga

**Affiliations:** 1 Department of Pharmaceutical Chemistry, Faculty of Pharmacy, “Iuliu Hațieganu” University of Medicine and Pharmacy, 41 Victor Babeș Street, 400010 Cluj-Napoca, Romania; alexandru.sandor@elearn.umfcluj.ro (A.Ș.); marc.gabriel@umfcluj.ro (G.M.); ooniga@umfcluj.ro (O.O.); 2 Department of Pharmacognosy, “Iuliu Hatieganu” University of Medicine and Pharmacy, 12 Ion Creangă Street, 400010 Cluj-Napoca, Romania; ioniga@umfcluj.ro; 3 Department of Surgical Oncology, “Iuliu Hațieganu” University of Medicine and Pharmacy, 34-36 Republicii Street, 40015 Cluj-Napoca, Romania; tudor.eniu@umfcluj.ro

**Keywords:** quinazoline, EGFR, structure–activity relationship, 4-anilino-quinazoline, competitive inhibitor, covalent inhibitor, allosteric inhibitor

## Abstract

The epidermal growth factor receptor (EGFR) plays a critical role in the tumorigenesis of various forms of cancer. Targeting the mutant forms of EGFR has been identified as an attractive therapeutic approach and led to the approval of three generations of inhibitors. The quinazoline core has emerged as a favorable scaffold for the development of novel EGFR inhibitors due to increased affinity for the active site of EGFR kinase. Currently, there are five first-generation (gefitinib, erlotinib, lapatinib, vandetanib, and icotinib) and two second-generation (afatinib and dacomitinib) quinazoline-based EGFR inhibitors approved for the treatment of various types of cancers. The aim of this review is to outline the structural modulations favorable for the inhibitory activity toward both common mutant (del19 and L858R) and resistance-conferring mutant (T790M and C797S) EGFR forms, and provide an overview of the newly synthesized quinazoline derivatives as potentially competitive, covalent or allosteric inhibitors of EGFR.

## 1. Introduction

Cancer is one of the most studied public health problems in the modern world, yet it is still one of the leading causes of death among developed countries [1,2,3]. With multifactorial etiology counting both endogenous [4,5] and exogenous [6] factors as drivers for mutagenesis and carcinogenesis [7,8], tumoral cells often harbor hundreds of genomic alterations [9] that lead to uncontrolled proliferation, differentiation, and expansion [10,11]. “The hallmarks of cancer” has been defined by Hanahan and Weinberg as biological capabilities of the tumoral cell developed during the multistep process of tumor progression [12]. The distinctive features that lead to malignancy development are: sustained proliferative signaling, resistance to apoptosis, avoidance of growth suppressors, enabling replicative immortality, development of blood vessels through neoangiogenesis, invasion of the adjacent tissue and metastasis, reprogramming cellular metabolism, and avoidance of immune destruction [12,13,14].

Cytotoxic therapy has been for a long time the gold standard in the treatment of cancer [15]. The inconsistent therapeutic response and low safety profile led to the discovery of novel strategies to target cancer [16]. The development and widespread clinical trials of highly selective anticancer agents such as therapeutic antibodies [17], tyrosine kinase inhibitors (TKIs) [18], micro-RNA therapy [19], oncolytic viruses [20], or gene-editing therapies [21] have led to an expansion of the arsenal against various types of tumors.

Nitrogen-containing heterocycles are a valuable source of pharmacophores for the development of novel active molecules, and more than 75% of the Food and Drug Administration (FDA)-approved molecules contain in their structures N-heterocyclic moieties [22]. Small molecules bearing nitrogen-containing heterocycles are valuable scaffolds for the development of novel inhibitors of various cellular signaling biomolecules [22,23,24].

EGFR has been one of the most extensively studied tyrosine kinase receptors whose role in oncogenesis has been well established [25,26]. The quinazoline core has been one of the most used heterocycles in the development of novel EGFR TKIs and also as a building block for the development of small molecules with an increased selectivity [27,28] aspect that is further discussed in this review.

The main purpose of this review is to outline the general principles for the development of potent EGFR TKIs, exploring the impact of structural modulations in key positions of the quinazoline core on the anticancer effect. It is intended as a guide for the rational development of future generations of quinazoline-based EGFR TKIs that comprises both already established drug design principles and an overview of the experimental quinazoline series reported from 2017 to the present as EGFR TKIs. By performing a Scopus database search using the keywords “quinazoline AND EGFR AND synthesis” we identified an increased interest in this field in recent years (Figure S1—Supplementary Data) [29]. Due to the vast amount of knowledge in the field of quinazolines, we chose to focus on the main SAR principles, while the other important topics such as synthesis methods will be discussed in our future works. Therefore, we selected 42 experimental quinazoline series to be discussed in the following sections (that met the minimal inclusion criteria: in vitro or in silico evidence of an EGFR inhibition mechanism of action).

## 2. Epidermal Growth Factor Receptor (EGFR)

### 2.1. EGFR Implications in Cancer

EGFR (HER1) is a tyrosine kinase transmembrane receptor, a member of the ErbB family along with HER2, HER3, and HER4 [30], that plays a crucial role in cell cycle progression [31], differentiation [32], and cytoskeletal rearrangements [33]. Each member of the erythroblastosis oncogene B (ErbB) family consists of an extracellular ligand-binding domain (ectodomain), a hydrophobic transmembrane domain, and an intracellular fragment with a tyrosine kinase domain that possesses enzymatic activity [34]. Signal transduction is generated by ligand binding in the extracellular domain, followed by dimerization and trans-autophosphorylation of various tyrosine residues in the C-tail of the receiving receptor that act as docking sites for various transductor proteins [35,36].

EGFR is one of the most extensively studied receptors due to its role in oncogenesis [37]. The proto-oncogenic role of EGFR has been established in several types of cancers, such as non-small-cell lung cancer (NSCLC) [38], glioblastoma [39], squamous cell carcinoma of the head and neck [40], breast cancer [41], colorectal cancer [42], and pancreatic cancer [43]. Oncogenic transformation of EGFR in tumor cells can lead to angiogenesis [44], accelerated cell proliferation, inhibition of apoptosis [31], cell motility, and metastasis [45,46]. Hyperactivation of EGFR signaling mediates intracellular antiapoptotic and pro-survival signals through downstream targets such as phosphatidylinositol-3 kinase–Akt (PI3K-AKT), mitogen-activated kinase (ERK), and signal transducer and activator of transcription proteins 3 (STAT3), making the survival of these cells EGFR-dependent [47,48,49].

EGFR signaling alteration is often associated with gene amplification, in-frame deletions, and specific mutations. Mutations often occur in specific locations mainly around the kinase domain, ectodomain, and C-terminal tail [50]. Extensive studies of EGFR signaling in NSCLC have revealed that most mutations occur around the kinase domain in around 30% of adenocarcinoma cases and promote constitutive activation in a ligand-independent manner [37,46]. The development of common mutations (L858R) leads to increased affinity for ATP by up to 20-fold that also impacts the affinity for small molecule inhibitors [35,51]. This makes EGFR an attractive target in the development of novel therapeutic agents as potent anticancer drugs for the treatment of NSCLC [52]. In other types of cancer, such as glioblastoma, ectodomain mutations (EGFR^vIII^ mutant found in 20% of the cases) can display ligand-independent signaling, low constitutive activity, and decreased down-regulation of endocytosis [39,53]. Increased gene amplification has been reported in several cases of colorectal cancer as an independent prognostic marker for anti-EGFR monoclonal antibodies [54].

### 2.2. EGFR Inhibition in Cancer

The EGFR inhibition of EGFR-dependent cancer cells leads to cell cycle arrest in the G1/S phase before DNA synthesis [55] by up-regulation of p^27KIP1^ activity [56,57] and up-regulation of pro-apoptotic molecules, such as Bcl-2-interacting mediator of cell death (BIM) that activates the intrinsic mitochondrial apoptotic pathway and cell death [58,59]. The blockage of EGFR signaling has been achieved by various strategies, among which two well-identified emerging categories of drugs have an established role in clinical practice: therapeutic antibodies, acting as competitive antagonists in the ectodomain [60], and TKIs, that disrupt the enzymatic activity of the kinase domain [18]. Other strategies such as immunotoxin conjugates [61], RNA-based therapeutics [62], vaccines [63], and protein degraders (PROTAC derivatives) [64] are still under clinical development. The following sections are mostly focused on the preclinical development and structure–activity relationship studies of EGFR TKIs in the last 6 years.

## 3. First Generation of EGFR TKIs

In-frame deletions in exon 19 (delE746_A750 or del19) and L858R insertion in exon 21, referred to as “common mutations”, are the oncogenic drivers for the development of more than 90% of the reported NSCLC cases found positive for a mutation of EGFR, reported with an increased incidence in Asian non-smoker females with a late-stage adenocarcinoma malignancy [65,66,67]. First-generation EGFR TKIs were specifically developed to inhibit the activation of common mutations bearing EGFR by replacing the adenosine triphosphate (ATP) in the catalytic site of the kinase in a competitive fashion and concentration-dependent manner [26].

**Figure 1:**
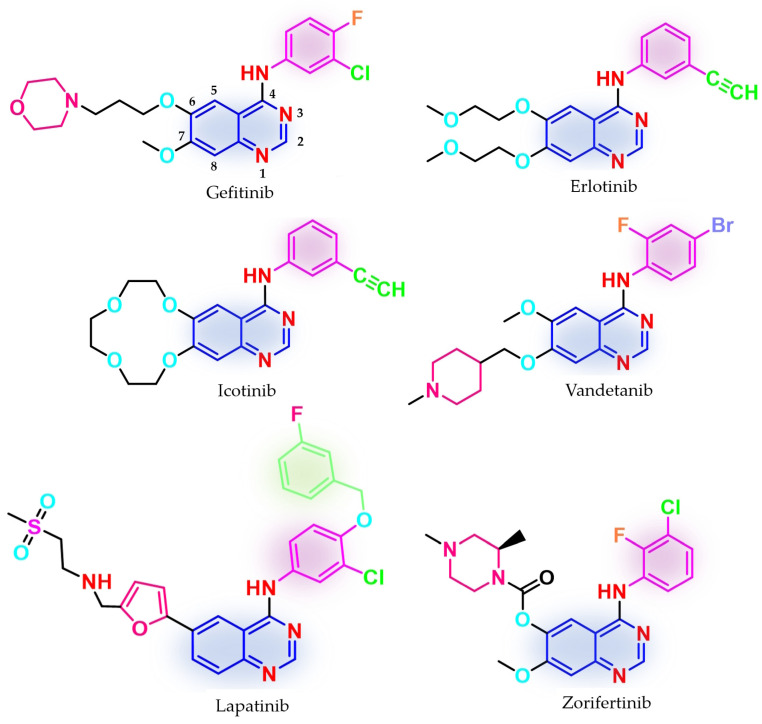
Chemical structures of first-generation quinazoline-based EGFR inhibitors approved in therapy and under clinical trial: gefitinib, erlotinib, icotinib, lapatinib, vandetanib, and zorifertinib.

Three first-generation inhibitors bearing a quinazoline core have been approved (Figure 1) for the treatment of NSCLC: gefitinib in 2003 (ZD1839, Iressa^®^—approved worldwide as first-line monotherapy for adults with locally advanced or metastatic NSCLC with del19 or L858R mutations detected by an FDA-approved test) [68,69,70]; erlotinib in 2004 (OSI-774, Tarceva^®^—approved for locally advanced or metastatic NSCLC with no improvement after four cycles of platinum-based chemotherapy and first-line therapy for locally advanced, complicated, or metastatic pancreatic cancer, combined with gemcitabine) [71,72,73]; and icotinib (approved in China for advanced or metastatic NSCLC) [74,75]. Phase III clinical trial IRESSA Pan-Asia Study (IPASS) demonstrated an increased period-free survival (PFS) for gefitinib compared to standard carboplatin/paclitaxel first-line therapy in an EGFR-mutation-positive group, higher objective response rate (ORR) (71.2% vs. 47.3%) [65,67], increased health-related quality of life index (HRQoL) [67,76], but no improvement in the overall survival (OR) (18.1 months vs. 18.3 months) [65,67]. Newer studies revealed that first-generation inhibitors are active on the L858R/C797S-harboring mutant following first-line treatment with osimertinib, a third-generation EGFR TKI, due to a non-covalent inhibition mechanism [77]. The main issue of the first-generation inhibitors is the development of secondary resistance-conferring mutations after a median period of 12 months by the replacement of Thr790 gatekeeper residue, which regulates access to the allosteric pocket, with a bulkier methionine [78,79,80]. Primary resistance was identified in patients harboring uncommon mutations (G719S, L861Q, S768I, and exon 20 insertions) [81,82,83,84]. In vitro studies suggest a 50-fold weaker affinity of gefitinib for EGFR^G719S^ compared to EGFR^L858R^ and a 14-fold higher affinity for ATP compared to EGFR^wt^ which makes those mutations less sensitive to first-generation inhibitors [51].

Other quinazoline-based TKIs, such as lapatinib (dual inhibitor of EGFR/HER2, approved in 2007 and used in association with capecitabine or letrozole for HER2-overexpressed advanced or metastatic breast cancer) [85,86] and vandetanib (pan-inhibitor of rearranged during transfection (RET), vascular epidermal growth factor receptor (VEGFR2 and VEGFR3), and EGFR, approved in 2011 for the treatment of unresectable, metastatic, or locally advanced medullary thyroid cancer) [87,88], whose therapeutic effects were proven to be associated with the inhibition of other targets [89], have emerged as models for the structural development of novel EGFR TKIs (Figure 1). Clinical trials are still underway for novel quinazoline derivatives such as zorifertinib, a first-generation EGFR TKI with increased lipophilicity and effectiveness in NSCLC with leptomeningeal metastasis (Figure 1) [90].

The molecular architecture of the ATP-binding pocket can be subdivided into specific regions according to the interactions between ATP and the key amino acids: hinge regions (adenine binding pocket) and solvent-accessible regions (sugar and phosphate binding region). Of particular interest for drug discovery are a series of hydrophobic pockets (I and II) and the back pocket (also called allosteric pocket) in the back of the gatekeeper residue that is in the proximity of the first mentioned regions [91,92,93]. Most of the approved quinazoline derivatives (gefitinib and erlotinib) follow a type I inhibition mechanism, binding to the active conformation of the receptor in a competitive manner with both ATP and Asp residue of the DFG-motif (Asp855-Phe656-Gly857) pointing inward to the binding site (αC-in conformation) [94], that narrows the efficiency spectrum of the EGFR mutants inhibited to activator mutations of the kinase domain [95]. Selected inhibitors (such as lapatinib) follow a type ½I inhibitory mechanism, binding mainly to the active conformation (αC-out and DFG-in) with access to the allosteric pocket and improved (but not clinically relevant) affinity for EGFR mutant forms stabilized in the inactive conformation [92,95].

**Figure 2:**
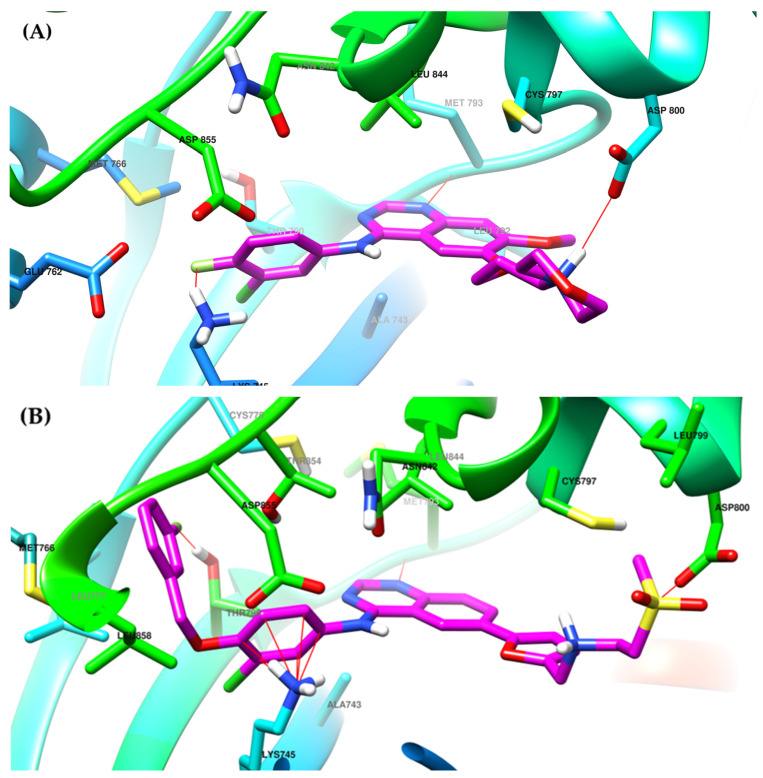

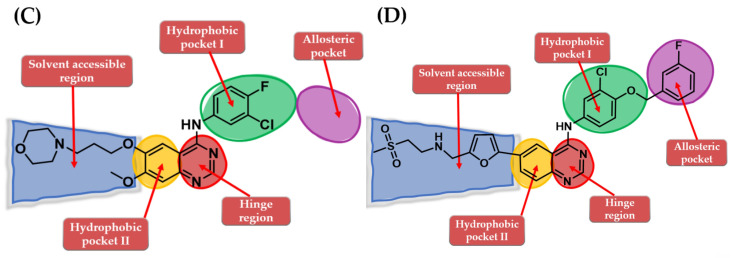
Visualization of gefitinib (**A**) and lapatinib (**B**) binding to the human EGFR kinase domain co-crystallized in the complexes 1XKK [101] and 2ITZ [51], from the Protein Data Bank [102] was performed using Chimera 1.10.2 (University of California, Oakland, CA, USA) [103]; regions of the active site and adjacent regions of the kinase domain occupied by gefitinib (**C**) and lapatinib (**D**).

The interactions of gefitinib in the active site of EGFR mutant L858R (Figure 2A,C) revealed that the quinazoline core is oriented in the back of the hinge region N-1 forming a hydrogen bond (H-bond) with the main amine chain of Met793. The 4-aniline moiety extends into the back of the ATP-binding cleft in the hydrophobic pocket forming a 45° angle with the quinazoline ring. The *meta*-chlorine and *para*-fluorine substituents increase the affinity through hydrophobic interactions. C-6 and C-7 extend into the solvent-accessible area forming an additional ionic bond through the tertiary amine of the morpholine moiety and Asp800. Erlotinib interacts with the active site of EGFR in a similar fashion, forming an H-bond in the hinge with Met769 at N-1 of the quinazoline ring and an extra H-bond through a water bridge with Gln767, Thr766, and Thr830 [96]. Lapatinib interacts differently with the residues in the active site (Figure 2B): while it maintains the H-bond with Met793, ionic bond with Asp800, and hydrophobic interactions at the aniline moiety, the presence of the (3-fluorophenyl)-methoxy moiety leads to novel interactions with the allosteric pocket and Thr790 (Figure 2D) compared to gefitinib.

The 4-anilino-quinazoline moiety has emerged as a privileged scaffold for developing novel first-generation EGFR TKIs due to a series of structural properties [27,97]. SAR studies of the already approved quinazoline-based EGFR TKI and novel synthesized compounds revealed that the formation of H-bonds between the N-1 quinazoline ring and a series of methionine residues (Met793 and Met769) and between N-3 and threonine residues through water bridges (Thr766 and Thr830) leads to a tighter binding conformation of the inhibitor to the active site of the kinase and also to increased potency [28,96,98].

The further substitution of the quinazoline ring in position 3 led to decreased activity through the steric hindrance of the H-bonds mentioned above. The presence of the aniline moiety [97] or another lipophilic moiety [27,99,100] in position 4 of the quinazoline core is mandatory for the affinity toward the ATP-binding pocket due to the extension in the hydrophobic pocket I and the generation of novel interactions unavailable to the ATP molecule [93,97]. The main focus in the development of novel first-generation EGFR TKIs is the further substitution of the aniline moiety and the replacement of the substituents in positions 6 and 7 [28], which will be discussed next.

## 4. Novel First-Generation Quinazoline EGFR TKIs (2017–Present)

### 4.1. 6,7-Dimorpholinoalkoxy-4-anilino-quinazolines

**Figure 3:**
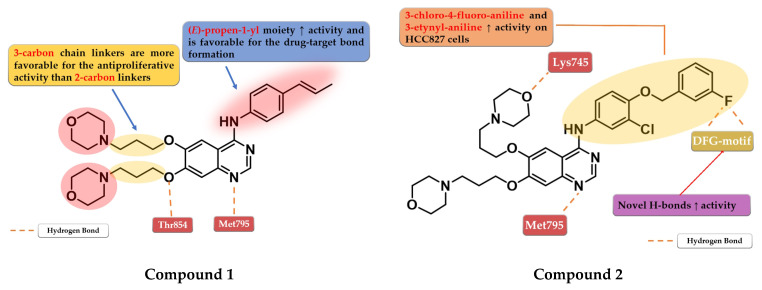
Structure–activity relationship and molecular docking of compound **1** in the human EGFR kinase domain (PDB ID 1XKK) [101], as reported by Chen et al. [104]; structure–activity relationship and molecular docking of compound **2** in the human EGFR kinase domain (PDB ID 1XKK) [101], as reported by Zhang et al. [105].

Chen et al. synthesized a series of 6,7-disubstituted 4-anilino-quinazoline derivatives with an (*E*)-propen-1-yl moiety as potent EGFR inhibitors. The compounds were evaluated for antiproliferative activity on four cell lines (A431, A549, NCI-H1975, and SW480). The newly synthesized compound **1** (Figure 3) showed significantly higher antiproliferative activity on all the cells than the reference drug gefitinib, and higher inhibition against EGFR^wt^ kinase than the reference drug lapatinib (20.72 nM vs. 27.06 nM) (Table S1—Supplementary Data). Insertion of morpholine alkoxy groups in positions 6 and 7 led to increased antiproliferative activity, with a 7.5-fold higher activity for the compounds with three-carbon linkers compared to shorter two-carbon chains. The insertion of (*E*)-propen-1-yl group in the *para*-position of the aniline moiety led to a different binding mode of compound **1** in the active site of EGFR and the formation of a supplementary H-bond with Thr854 [104].

Based on these results, Zhang et al. synthesized a series of novel 6,7-dimorpholinoalkoxy quinazoline derivatives with moderate to high antiproliferative activity. The compounds bearing a three-carbon chain linker between the quinazoline and morpholine cores were found more potent against the A431 cell line than the ones bearing a carbon chain of two atoms and the reference drugs, gefitinib and lapatinib. A longer chain allows the formation of an H-bond between the oxygen of the morpholine and the Lys745 residue of the kinase domain, leading to higher affinity toward the target. The most active compound **2** (Figure 3) is a 3-chloro-4-(3-fluorobenzyloxy) aniline derivative, with higher antiproliferative activity than those of the reference drugs on all the five cell lines and elevated inhibitory activity toward a large panel of mutated kinases (Table S1). The presence of the two morpholine alkoxy substituents in positions 6 and 7 of the quinazoline core determines a shift in the binding mode compared to lapatinib, leading to novel H-bond interactions between the fluorine residue and Asp855/Phe856 of the DFG-motif [105].

### 4.2. 6-Aryl-semicarbazone-4-anilino-quinazoline Derivatives

**Figure 4:**
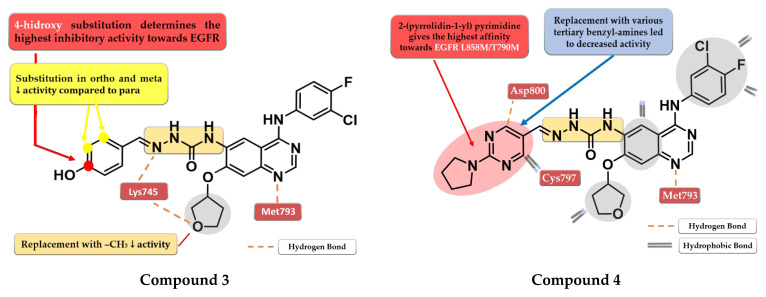
Structure–activity relationship and molecular docking of compound **3** in the human EGFR^T790M^ kinase domain (PDB ID 4G5J) [108], as reported by Tu et al. [106]; structure–activity relationship and molecular docking of compound **4** in the human EGFR^T790M^ kinase domain (PDB ID 4G5J) [108], as reported by Wang et al. [107].

A novel series of quinazoline derivatives has been synthesized by Tu et al. by inserting an arylidene-semicarbazone moiety in the C-6 position of the lead compound afatinib. Remarkable results have been obtained with the 4-hydroxy substitution of the phenyl ring, the most active compound **3** (Figure 4) showing higher antiproliferative activity (HepG2—0.07 nM, MCF—0.91 nM, and PC-3—0.91 nM) than the reference drug afatinib (HepG2—1.40 nM, MCF—2.63 nM, and PC-3—2.62 nM) on three out of four cell lines in in vitro studies (Table S1). The replacement of the 4-hydroxy group with hydrophobic substituents (-F, -Cl, and -Br) or the further *ortho*- and *meta*-substitution with hydrophobic or hydrophilic (-OH and -OCH_3_) groups on the 4-hydroxy-phenyl moiety led to decreased activity [106]. The low potency of compound **3** toward the mutant form EGFR^L858R/T790M^ (IC_50_ > 1000 nM) and its low stability led to further structural optimization focusing on the phenolic moiety. The best results were achieved by the replacement of the benzene ring with nitrogen-containing heterocycles to generate novel H-bonds with the target. Compound **4** (Figure 4) bearing a 2-(pyrrolidin-1-bestpyrimidine side chain had the most promising results, with a similar antiproliferative and EGFR ^L858R/T790M^ inhibitory activity (IC_50_ = 8.4 nM) to that of the reference drug afatinib (IC_50_ = 3.8 nM) (Table S1) [107].

### 4.3. Disubstituted-Urea/Thiourea-Linked 4-Amino-quinazoline Derivatives

**Figure 5:**
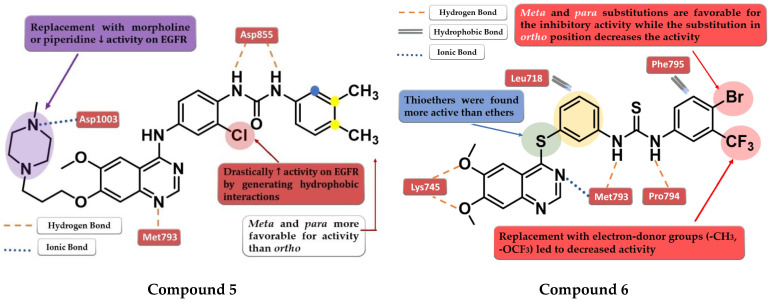
Structure–activity relationship and molecular docking of compound **5** in the human EGFR kinase domain (PDB ID 2ITY) [51], as reported by Zhang et al. [109]; structure–activity relationship and molecular docking of compound **6** in the human EGFR kinase domain (PDB ID 2ITY) [51], as reported by Sun et al. [110].

Zhang et al. synthesized a novel series of 4-anilino-quinazoline derivatives as dual inhibitors of EGFR and VEGFR2 by replacing the small hydrophobic substituents on the aniline with phenyl urea residues. The substitution of the terminal benzene ring in the *para* and *meta* positions with small hydrophobic substituents (-Cl and -CH_3_) led to elevated inhibitory activity toward both EGFR and VEGFR2, while the *ortho*-substitution led to less potent compounds. Further modification of the C-7 side chain residue concluded that the insertion of a 4-methyl-piperazine-containing residue gives the highest inhibitory activity toward EGFR due to the capability of generating an ionic bond with the carboxyl residues in the target’s structure. The replacement of the piperazine with morpholine or piperidine led to less potent EGFR inhibitors. The presence of the chloro-substituent in the *meta* position of the aniline residue further increased the inhibitory activity toward both EGFR and VEGFR2. The most active compound **5** (Figure 5) was an 11-fold more potent EGFR inhibitor (IC_50_ = 1 nM) compared to the reference drug vandetanib (IC_50_ = 11 nM) and had higher antiproliferative activity on all the tested cells (Table S1) [109].

A novel series of diaryl-thiourea-linked quinazoline derivatives have been synthesized as dual EGFR/VEGFR2 inhibitors by Sun et al. The presence of two electron-withdrawing groups (EWGs) on the terminal benzene ring led to elevated inhibitory activity toward both EGFR and VEGFR2 and higher antiproliferative activity on all three tested cell lines (HCT-116, MCF-7, and B16). The substitution with electron-donating groups (EDGs) (-CH_3_ and -OCF_3_) led to decreased inhibitory activity against EGFR/VEGFR2 kinases and antiproliferative activity. The most active compound **6** (Figure 5) displayed remarkable inhibitory activity against EGFR (IC_50_ = 10 nM) and VEGFR2 (IC_50_ = 80 nM) compared to the reference drug sorafenib (20 nM and 80 nM), and better antiproliferative activity on all the tested cell lines (Table S1). In vivo tests revealed a two-fold increase in the inhibition of tumor growth in the xenograft model of B16 melanoma for compound **6** (64.04%) compared to sorafenib (31.25%). Docking studies of compound **6** in the active site of EGFR revealed a tight binding due to the multiple H-bonds formed by the thiourea moiety and the molecular tail, the low-electron-density areas of the molecule being engaged in hydrophobic interactions [110].

**Figure 6:**
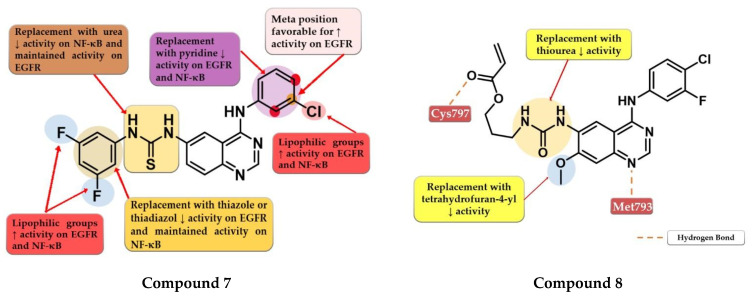
Structure–activity relationship of compound **7** as reported by Hamad et al. [111]; structure–activity relationship and molecular docking of compound **8** in the human EGFR^T790M^ kinase domain (PDB ID 4G5J) [108], as reported by Gan et al. [112].

Hamad et al. synthesized a series of novel dual inhibitors of EGFR kinase and nuclear factor kappa B (NF-κB) activities. The insertion of various hydrophilic substituents (sulfonamides, hydroxy groups) on the 4-anilino moiety or the total replacement with amino-pyridine led to decreased inhibitory activity for both EGFR and NF-κB. The substitution with small and lipophilic (-Br, -Cl, and -methyl) groups particularly in the *meta* position increased the inhibitory activity. The thiourea linker is mandatory for the inhibitory activity toward NF-κB. The replacement with a urea linker led to the decreased inhibitory activity of NF-κB but conserved affinity for EGFR kinase. The substitution of the lateral benzene ring at the C-6 position of the quinazoline core with small and lipophilic groups increased inhibition potency for both targets while the insertion of hydrophilic groups led to decreased activity. Total replacement of the lateral benzene ring with substituted thiadiazole or thiazole rings led to increased affinity toward NF-κB and less affinity for EGFR (>100 nM). In vitro data of the antiproliferative activity of prototype compounds “6c” and “6h” (structures not provided) on A549 cells (1.6 µM and 1.0 µM) suggested that the dual inhibitory mechanism might provide an advantage in EGFR-dependent cell lines. SAR studies led to the synthesis of compound **7** (Figure 6) with remarkable inhibitory activity toward NF-κB (IC_50_ = 0.3 µM) and EGFR kinase in a nanomolar range (IC_50_ = 60.1 nM). Compound **7** displayed elevated antiproliferative activity on MDA-MB-231 cells (0.9 µM), higher than that of the reference drug gefitinib (14.2 µM) (Table S1), and inhibited the in vivo development of a tumor in a xenograft model with fewer side effects [111].

Gan et al. synthesized a series of novel 4-arylamino-quinazoline derivatives as potential EGFR^T790M/L858R^ inhibitors. Preliminary studies revealed that urea linkers are more favorable for inhibitory activity compared to thiourea-containing derivatives. The most active compound **8** (Figure 6) displayed comparable inhibitory activity toward EGFR^wt^ (IC_50_ = 0.8 nM) and EGFR^T790M/L858R^ (IC_50_ = 2.7 nM) with that of the reference drug afatinib (0.6 nM and 3.5 nM) and broad-spectrum cytotoxic activity (H1975, A549, HeLa, and MCF-7 cells) (Table S1). Compound **8** acts in a concentration-dependent manner similar to first-generation inhibitors by forming only H-bonds with Cys797 through the ester group at the C-6 position and not covalent adducts [112].

### 4.4. 6-Substituted-amide-4-amino-quinazoline Derivatives

**Figure 7:**
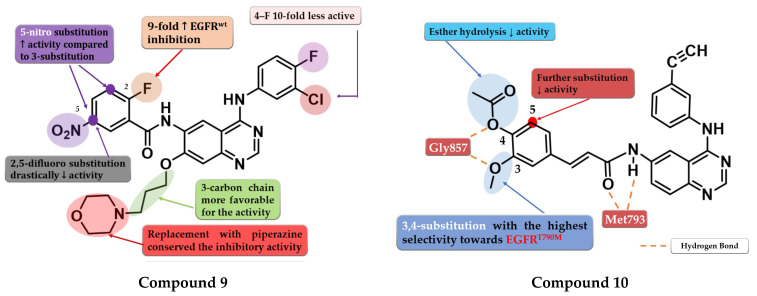
Structure–activity relationship of compound **9** as reported by Hou et al. [113]; structure–activity relationship and molecular docking of compound **10** in the human EGFR^T790M^ kinase domain (PDB ID 2JIU) [115], as reported by Zhang et al. [114].

A series of 6-benzamide quinazoline derivatives that reversibly inhibit the EGFR activity were synthesized by Hou et al. The introduction of a fluor-substituent in the C-2 position of the benzene ring is vital for inhibitory activity. Further substitution at the C-5 position of the benzamide moiety with a nitro group (-NO_2_) led to a two-fold increase in inhibitory activity on EGFR^wt^ kinase. Replacement of the amide linker with a methyl-amino linker between phenyl and quinazoline led to an almost 50-fold decrease in inhibitory activity. Bulkier substituents in position C-7 of the quinazoline core are favorable for inhibitory activity. The replacement of morpholine with piperazine or dimethylamine led to conserved activity. The most promising compound **9** (Figure 7) displayed high antiproliferative activity against various cells harboring a mutation of the EGFR, minimal toxicity on HEK293 cells and EGFR^wt^ bearing cells, high selectivity toward EGFR in kinase inhibition profiling (Table S1), and favorable pharmacokinetics [113].

To overcome the T790M mutation, Zhang et al. synthesized a series of novel cinnamamide-quinazoline derivatives. The highest affinity toward EGFR^T790M^ was achieved through the substitution of the lateral phenyl moiety with 3-methoxy and 4-acetoxy groups by generating a higher selectivity profile and favorable pharmacokinetics. The further substitution of the lateral benzene ring, 3-acetoxy/4-methoxy substitution profile, and the hydrolysis of the ester group led to a decrease or total loss of antiproliferative activity on all three tested cell lines (A431, A549, and H1975). The most active compound **10** (Figure 7) displayed comparable antiproliferative activity on H1975 cells (1.22 µM) with reference drug osimertinib (11.29 µM) but a lower selectivity index (2.72 vs. 4.6) (Table S1) [114].

**Figure 8:**
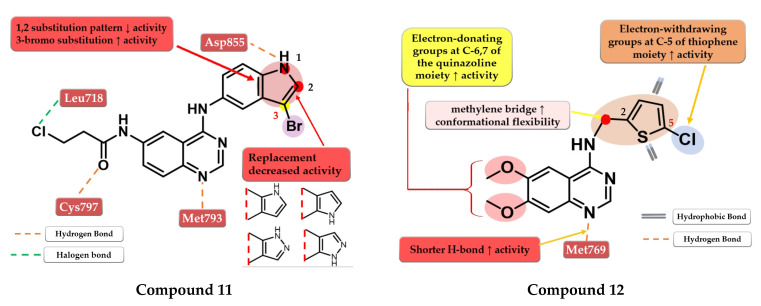
Structure–activity relationship and molecular docking of compound **11** in the human EGFR^T790M^ kinase domain (PDB ID 4G5J) [108], as reported by Tang et al. [116]; structure–activity relationship and molecular docking of compound **12** in the human EGFR^T790M^ kinase domain (PDB ID 4HJO) [96], as reported by Zou et al. [117].

Tang et al. developed a novel series of reversible/irreversible pan-HER inhibitors bearing an N-(3-bromo-1H-indol-5-yl)-quinazolin-4-amine scaffold. Preliminary SAR studies revealed that the insertion of 1H-indol-5-amine in position 4 of the quinazoline core led to sub-nanomolar IC_50_ values. The further substitution of the indole moiety with various substituents concluded that the in positions 1 and 2 led to decreased in vitro activity while the insertion of a small lipophilic bromine in position 3 gave the best results. These data determined the synthesis of the most active compound **11** (Figure 8) as a pan-HER high-strength reversible inhibitor displaying comparable inhibitory activity toward EGFR^wt^ (IC_50_ = 0.38 nM) and EGFR^T90M/L858R^ (IC_50_ = 2.2 nM) with the reference drug afatinib (IC_50_ = 0.67 nM and IC_50_ = 3.7 nM). A multi-kinase assay revealed that compound **11** is also a potent HER2 and HER4 inhibitor. The compound displayed increased cytotoxic activity on high-expression EGFR cells (A431, H1975, and HCC82) and HER2 (SK-BR-3 and BT-474) similar to the reference drug afatinib (Table S1) [116].

### 4.5. 6.7-Dimethoxy-4-amino-quinazoline Derivatives

Zou et al. synthesized a series of novel quinazoline derivatives by inserting a thiophene-2-ylmethanamine at the C-4 position of the quinazoline core to increase the conformational flexibility. The synthesized molecules displayed good antiproliferative activity on A431 cells, the most active compound **12** (Figure 8) having comparable results (IC_50_ = 3.4 µM) with the reference drug erlotinib (IC_50_ = 3 µM) (Table S1). The insertion of electron-donating groups at the 6 and 7 positions of the quinazoline core increased the activity of the compounds. The 6,7-dimethoxy substitution was more favorable for the inhibition of EGFR due to the slight deviation of the quinazoline core in the active site of EGFR generated by bulkier groups (2-methoxy-ethyl-oxy) leading to shorter H-bonds with Met769 and the N-1 of the quinazoline core. The further substitution of the C-5 position of the thiophene moiety with small lipophilic substituents (-Cl and -Br) led to a significant increase in the antiproliferative activity [117].

### 4.6. 6-Heteroaryl-4-amino-quinazoline Derivatives

**Figure 9:**
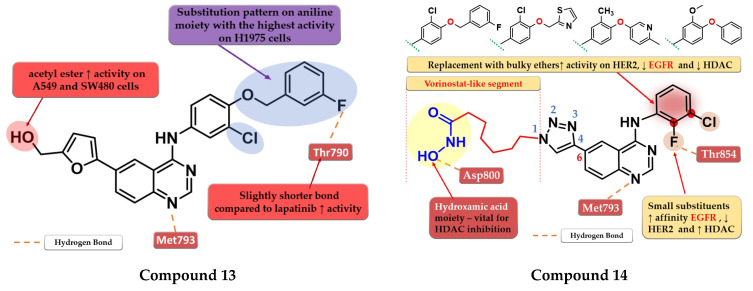
Structure–activity relationship and molecular docking of compound **13** in the human EGFR kinase domain (PDB ID 1XKK) [101], as reported by Zhang et al. [118]; structure–activity relationship and molecular docking of compound **14** in the human EGFR kinase domain (PDB ID 1XKK) [101], as reported by Ding et al. [119].

A novel series of 4-anilino-quinazoline derivatives bearing a 5-substituted furan-2-yl moiety at the C-6 position of the quinazoline core was synthesized by Zhang et al. The most active compound **13** (Figure 9) displayed a two-fold higher antiproliferative activity on A549 cells (7.35 µM) compared to the reference drugs gefitinib (21.17 µM) and lapatinib (14.09 µM), and significantly higher antiproliferative activity on H1975 cells (3.01 µM, 9.08 µM, and 8.05 µM) (EGFR^L858R/T790M^) (Table S1). The acetyl-derivative of compound **13** displayed similar antiproliferative activity to that of compound **13** on SW480 and A549 cells but was found to be less potent on the other two cell lines. Compound **13** was found to be a highly potent EGFR^wt^ inhibitor in vitro (IC_50_ = 5.06 nM) with a significantly higher activity compared to lapatinib (IC_50_ = 27.06 nM) and similar activity to gefitinib (IC_50_ = 3.22 nM) (Table S1). The docking studies of compound **13** in the active site of EGFR revealed a binding mode similar to that of lapatinib, with the five-fold difference in activity being correlated with shorter and stronger H-bonds generated [118].

By merging the 4-anilino-quinazoline structural motif and a hydroxamic acid moiety, Ding et al. synthesized a series of novel 6-(1,2,3-triazol-4-yl)-4-amino-quinazoline derivatives with multitarget inhibitory activity, including EGFR, HER2, histone deacetylases 1 (HDAC1), and 6 (HDAC6). Smaller and lipophilic substituents (-F and -Cl) on the aniline moiety led to enhanced affinity toward EGFR, moderate activity toward HER2, and higher potency toward both HDAC1/HDAC6 compared to the reference drug vorinostat. The substitution with various bulky ethers (3-fluorobenzyloxy, thiazol-2-ylmethoxy, 6-methylpyridinyl-3-oxy, and 3-methoxy-4-phenoxy) of the aniline moiety increased activity on HER2, decreased inhibitory activity for EGFR by more than 10-fold and drastically reduced the activity on HDAC1/HDAC6 due to the steric hindrance on the outside surface of the HDAC1. The insertion of the vorinostat-like segment at the C-6 of the quinazoline core led to a higher affinity toward EGFR compared to HER2. The most active compound **14** (Figure 9) displayed higher antiproliferative activity on A549 cells (0.63 µM) and lower antiproliferative activity on BT-474 cells (3.88 µM) compared to the reference drugs lapatinib (1.74 µM and 0.1 µM) and vorinostat (2.57 µM and 2.67 µM) (Table S1). The compound displayed a similar antiproliferative activity pattern on A431 cells (EGFR overexpressed) and SK-BR-3 (HER2 overexpressed) while showing lower activity on H1975 cells compared to lapatinib, which could be correlated with the lower affinity toward HER2 and therefore blocking the heterodimerization of EGFR-HER2 to a lesser extent (Table S1) [119].

### 4.7. Nitro-Substituted-Azole Linked 4-Amino-quinazoline Derivatives

**Figure 10:**
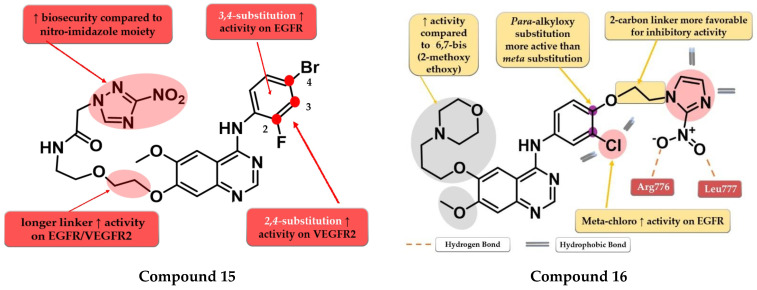
Structure–activity relationship and molecular docking of compound **15** in the active site of EGFR as reported by Wei et al. [120]; structure–activity relationship and molecular docking of compound **16** in the human EGFR kinase domain (PDB ID 1XKK) [101], as reported by Cheng et al. [121].

By inserting the 3-nitro-1,2,4-triazole motif at the C-7 position of the quinazoline core Wei et al. synthesized a series of novel 4-anilino-quinazoline derivatives as dual EGFR/VEGFR2 inhibitors active in hypoxic conditions. A longer chain linker between the quinazoline core and the triazole moiety was found to be favorable for the inhibitory activity toward both EGFR and VEGFR2. Most of the synthesized compounds displayed increased inhibitory activity toward EGFR (IC_50_ = 0.37 to 12.93 nM). A 2,4-substitution on the aniline moiety with heavy and bulky halogen atoms increased the activity on VEGFR2 while a 3,4-substitution increased activity on EGFR even more. Compound **15** (Figure 10) displayed a 3.5-fold higher inhibitory activity on EGFR (5.9 nM) and similar inhibitory activity on VEGFR2 (36.78 nM) compared to the reference drug vandetanib (19.76 nM and 33.26 nM). It also displayed significantly higher antiproliferative activity on both hypoxic and hypoxic conditions associated with irradiation on the tested cell lines (A549 and H446) compared to vandetanib (Table S1) and remarkable inhibition on A549 tumor growth in the in vivo model [120].

Cheng et al. synthesized a series of lapatinib analogs by replacing the benzyloxy moiety with nitro-imidazole residues to obtain compounds with increased activity in hypoxic conditions. Compound **16** (Figure 10) displayed higher antiproliferative activity on the tested cells in both normoxia (1.59 µM and 2.46 µM) and hypoxia (1.09 µM and 1.35 µM) conditions compared to the reference drug lapatinib (11.3 µM and 13.26 µM; 6.81 and 8.85 µM) (Table S1). The *meta*-chlorine substitution on the aniline moiety was beneficial for the activity due to the hydrophobic interactions. The insertion of 2-nitro-imidazole-1H-alkyloxyl in the *para* position of the aniline moiety led to significantly higher activity on EGFR compared to *meta* substitution, generating a binding mode in the active site of EGFR similar to lapatinib. Compounds bearing a three-carbon alkyloxy linker were less potent than the ones bearing a two-carbon bridge due to an unfavorable binding mode within the active site of EGFR leading to a more “out” binding position compared to lapatinib [121].

**Figure 11:**
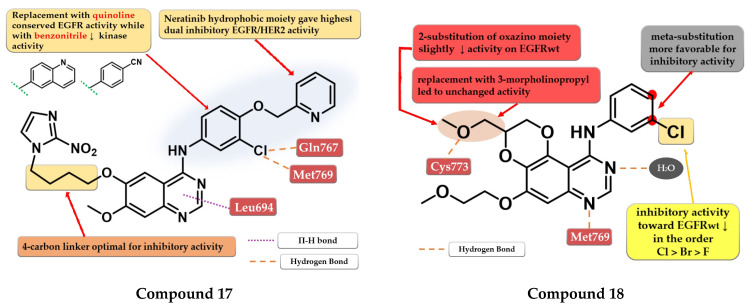
Structure–activity relationship and molecular docking of compound **17** in the human EGFR kinase domain (PDB ID 1M17) [124], as reported by Elkamhawy et al. [122]; structure–activity relationship and molecular docking of compound **18** in the human EGFR kinase domain (PDB ID 1M17) [124], as reported by Quin et al. [123].

Elkamhawy et al. developed a series of 4-anilino-quinazoline derivatives as dual EGFR/HER2 that incorporate the nitro-imidazole moiety active in hypoxic conditions. SAR studies revealed that the optimal length of the carbon chain linker for the highest dual inhibitory activity is four. The most active compound **17** (Figure 11) was synthesized by inserting the hydrophilic moiety of neratinib (structure not provided) in position 4 of the quinazoline core and displayed an increased inhibitory activity toward EGFR kinase (1.8 nM) compared to the reference drugs (lapatinib–10 nM and staurosporine–88.1 nM) and significantly less affinity toward HER2 kinase (Table S1). Replacement of the (3-chloro-4-(pyridin-2-ylmethoxy)-aniline moiety with quinoline led to conserved EGFR activity while the substitution with benzonitrile led to activity reduction on both kinases [122].

### 4.8. Fused 2,3-Dihydro-[1,4] Dioxino-[2,3-f] Quinazoline Derivatives

Quin et al. synthesized a series of novel 2,3-dihydro-[1,4]-dioxino-[2,3-f]-quinazoline derivatives as EGFR inhibitors. The cyclization at C-5 and C-6 of the quinazoline core led to smaller and more compact molecules, able to tolerate variations in the structure of the kinase domain due to secondary or tertiary mutations. The substitution of the aniline moiety with small and hydrophobic substituents (decreased activity in the order F < Br < Cl) increased the activity compared to smaller EDGs (25). The most active compound **18** (Figure 11) displayed antiproliferative activity (A549–9.95 µM and NCI-H15-11.66 µM) comparable to the reference drug erlotinib (7.26 µM and 6.88 µM), and inhibitory activity toward EGFR^wt^ kinase (10.29 nM) similar to reference drugs gefitinib and erlotinib (10.41 nM and 11.65 nM) (Table S1) [123].

### 4.9. (2-Bromo-phenyl)-4-amino-quinazoline Derivatives

**Figure 12:**
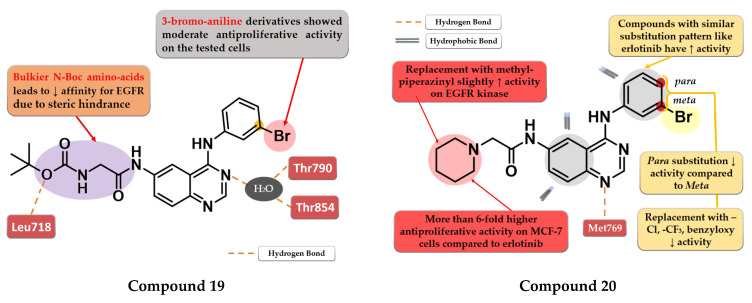
Structure–activity relationship and molecular docking of compound **19** in the human EGFR kinase domain (PDB ID 2ITY) [51], as reported by Zheng et al. [125]; structure–activity relationship and molecular docking of compound **20** in the human EGFR kinase domain (PDB ID 1XKK) [101], as reported by Ismail et al. [126].

Zheng et al. evaluated the addition of various N-Boc amino acid moieties in position 6 of the 4-anilino-quinazoline scaffold on antiproliferative activity. The 3-bromo-aniline compound **19** (Figure 12) bearing an N-Boc glycine residue in position 6 showed moderate antiproliferative activity on HepG2 cells (IC_50_ = 8.3 µM) and a potent inhibitory activity against EGFR (IC_50_ = 3.2 nM) with a 2000-fold higher selectivity over various kinases related to liver cancer (Table S1). Molecular docking studies of compound **19** revealed a novel H-bond interaction between the nitrogen atom of the side chain in position 6 and Leu718. The steric hindrance of this bond by replacing N-Boc glycine with various bulkier N-Boc amino acids led to decreased activity [125].

Ismail et al. synthesized a series of 4-anilino-quinazoline derivatives by attaching 2-substituted acetamido moieties at the C-6 of the quinazoline core. The most active compound **20** (Figure 12) displayed potent antiproliferative activity on both tested cell lines (HepG2 12 µM and MCF-7 3 µM) compared to the reference drug erlotinib (25 µM and 20 µM) (Table S1). *Meta*-bromoaniline derivatives displayed higher antiproliferative activity compared to *para*-substituted derivatives, especially when associated with piperidinyl moiety on the acetamide spacer at the C-6 position. The further replacement of -Br with -Cl, -CF_3_, or benzyloxy group led to decreased activity. A possible explanation is that the substitution pattern on the aniline moiety could be correlated with the results of the docking studies which indicated that the most active compounds presented a binding mode similar to erlotinib [126].

### 4.10. 2-Aryl-4-Substituted Quinazoline Derivatives

**Figure 13:**
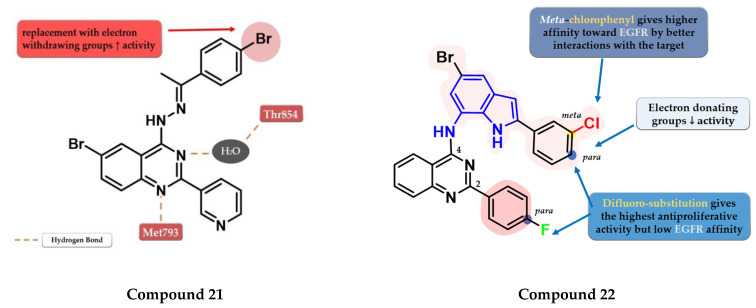
Structure–activity relationship and molecular docking of compound **21** in the human EGFR kinase domain (PDB ID 1XKK) [101], as reported by Ahmed et al. [127]; structure–activity relationship of compound **22**, as reported by Mphahlele et al. [128].

Ahmed et al. synthesized a novel series of 6-bromo-2-(pyridin-3-yl)-4-substituted quinazolines as potential dual EGFR-HER2 inhibitors. The 4-bromo-phenylethylidene-hydrazinyl compound **21** (Figure 13) displayed the highest inhibitory activity toward EGFR^wt^ (46.1 nM) comparable with that of lapatinib (53.1 nM). The *para*-substitution of the phenyl moiety with various EWGs (-Cl, -Br, -F, and -SO_2_CH_3_) led to higher inhibitory activity compared to electron-donating groups (-CH_3_ and -OCH_3_). In vitro testing revealed an increased cytotoxic activity toward MDA-MB-231 cells compared to the reference drug lapatinib (Table S1) [127].

Mphahlele et al. synthesized a series of indole-amino-quinazolines with various substituents in positions 2 and 4 of the quinazoline core and evaluated their antiproliferative activity on 5 different cell lines (A549, Caco-2, C3A, MCF-7, and HeLa). The presence of small lipophilic substituents in *para* (-F) on both side benzene rings in positions 2 and 4 of the quinazoline core led to increased antiproliferative activity on all the tested cells, while the substitution with *para*-chlorophenyl on the indole moiety and unsubstituted benzene at position 2 led to potent selective toxicity on A549 and Caco-2 cells (structures not provided). The replacement of the halogen hydrophobic substituents with EWGs (-OCH_3)_ led to a major decrease in the activity on all the cells. The highest affinity for EGFR was identified for compound **22** (IC_50_ = 40.7 nM) (Figure 13) which showed similar EGFR inhibitory activity compared to gefitinib (IC_50_ = 38.9 nM) and moderate antiproliferative activity (Table S1) [128].

### 4.11. Sulfonamide Linked Quinazoline Derivatives

**Figure 14:**
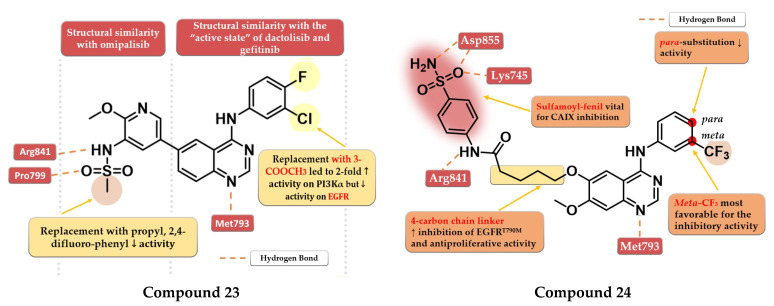
Structure–activity relationship and molecular docking of compound **23** in the human EGFR kinase domain (PDB ID 4WKQ) [131], as reported By Ding et al. [129]; structure–activity relationship and molecular docking of compound **24** in the human EGFR^T790M^ kinase domain (PDB ID 2JIU) [115], as reported by Zang et al. [130].

A series of dual EGFR-PI3Kα inhibiting 4-amino-quinazoline derivatives were synthesized by Ding et al. by merging the methoxy-pyridil-3-yl-sulfonamide moiety (found in PI3Kα-specific inhibitor omipalisib—structure not provided) and 4-arylamino-quinazoline motif (Figure 1). Methyl-sulfonamide derivatives were more active than propyl or phenyl-substituted sulfonamide derivatives due to the steric hindrance and lack of new H-bond interactions in the sulfonamide group. The replacement of aniline residue at the C-4 position with 3-amino-pyridine led to conserved activity. The most active compound **23** (Figure 14) displayed similar inhibitory activity on the EGFR kinase to that of the reference drug gefitinib (IC_50_ = 2.4 nM) and less potent affinity toward PI3Kα (IC_50_ = 317 nM) compared to the reference drug dactolisib (IC_50_ = 16.4 nM) as well as moderate cytotoxic activity (Table S1) [129].

Zhang et al. synthesized a series of novel EGFR/carbonic anhydrase IX (CAIX) dual inhibitors by merging the sulfamoyl-aryl moiety found in various CAIX inhibitors with the 4-anilino-quinazoline scaffold. The *meta* substitution of the aniline ring with the trifluoromethyl group gave the highest antiproliferative activity on all cells (A549, A431, and H1975), while the insertion of a second substituent in *para* or mono-*para* substitution led to decreased activity. A flexible four-carbon linker between the sulfamoyl-aryl moiety and quinazoline core was found to increase the antiproliferative and inhibitory activities toward both EGFR^wt^ and EGFR^T790M^ kinase. The most active compound **24** (Figure 14) displayed higher antiproliferative activity on EGFR^wt^ positive cell lines (A549 6.54 µM and A431 4.04 µM) than the reference drug gefitinib (15.59 µM and 8.37 µM) and slightly less potent antiproliferative activity on EGFR^T790M^-positive cells (1.94 µM) compared to osimertinib (0.98 µM) (Table S1). Compound **24** is a highly specific inhibitor of EGFR (IC_50_ = 9.2 nM) with a comparable affinity to that of the reference drug osimertinib (IC_50_ = 8.1 nM) and lower inhibitory activity toward EGFR^wt^ (IC_50_ = 27 nM) compared to gefitinib (IC_50_ = 17.1 nM). The selectivity of compound **24** toward the mutant form of EGFR could be correlated with the multiple H-bonds generated by the sulfamoyl moiety in the solvent-accessible region of the mutant form of the kinase compared to just one H-bond in the EGFR^wt^ kinase [130].

### 4.12. (4-Cyano-phenyl)-4-amino-quinazoline Derivatives

**Figure 15:**
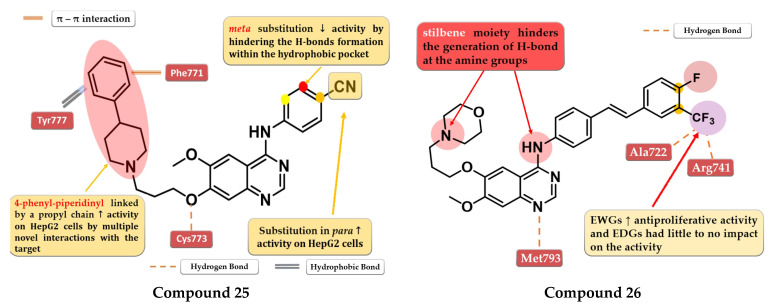
Structure–activity relationship and molecular docking of compound **25** in the human EGFR kinase domain (PDB ID 1M17) [124], as reported by Chang et al. [132]; structure–activity relationship and molecular docking of compound **26** in the human EGFR kinase domain (PDB ID 1XKK) [101], as reported by Wang et al. [133].

A series of 4-anilino-quinazoline derivatives were synthesized by Chang et al. by modifying the substituent in the C-7 position of the quinazoline core. Highly rigid substituents (methylbenzyl-amino, hydroxyethyl-benzylamino, and 2-hydroxyethyl-piperidinyl) and amino alcohol groups (4-hydroxypiperidinyl and 4-hydroxyethylpiperidinyl) linked in the C-7 position of the quinazoline core led to weak antiproliferative activity. Remarkable results were obtained with the 4-piperidinyl-piperidinyl, 4-phenylpiperidinyl, 4-ethyl-piperazinyl, and pyrrolyl residues. The most active compound **25** (Figure 15) has a broad anticancer effect, higher antiproliferative activity, and inhibitory activity toward EGFR similar to the reference drug gefitinib (3.62 nM) (Table S1). The binding mode of compound **25** into the kinase domain of EGFR showed multiple interactions of the C-7 side chain, including hydrophobic and π–π interactions with Phe771 and Tyr777 residues [132].

### 4.13. 6,7-Dialkoxy-4-stilbenyl-amino-quinazolines

A series of novel 4-stilbenyl-amino-quinazolines derivatives were synthesized by Wang et al. The substitution of the classical halogen-substituted anilines with (*E*)-4-styrylanilines at position 4 of the quinazoline core can lead to enhanced stability and lipophilicity while the further substitution of the stilbene moiety with EWGs (-F, -Br, and -CF_3_) augmented the antiproliferative activity. All the tested compounds showed high antiproliferative activity (IC_50_ = 1.23–5.17 μM) compared to the reference drug gefitinib on all the tested cells (A431 12.93 µM, A549 13.75 µM, and BGC-823 > 10 µM). The most active was the trifluoromethyl styryl compound **26** (Figure 15, Table S1). The presence of -CF_3_ in position 3 or 4 of the side benzene ring enhanced the antiproliferative activity by generating novel acceptor H-bonds with arginine and glycine in the hydrophobic pocket of the active site of EGFR [133].

### 4.14. Macrocyclic Quinazoline Derivatives

**Figure 16:**
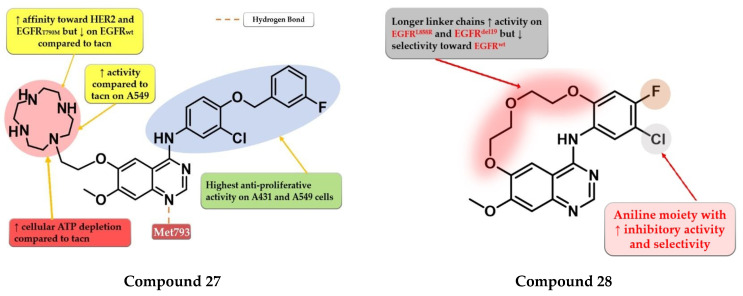
Structure–activity relationship and molecular docking of compound **27** in the human EGFR kinase domain (PDB ID 1XKK) [101], as reported By Ju et al. [134]; structure–activity relationship compound **28**, as reported by Amerheim et al. [135].

The low electron density of the morpholine core in the structure of gefitinib (Figure 1) led Ju et al. to synthesize a novel series of quinazoline derivatives with a macrocyclic polyamine side chain at the C-6 position of the quinazoline core. The replacement of the morpholine moiety with 1,4,7,10-tetraazacyclododecane (cyclen) or 1,4,7-triazonane (tacn) led to a two-fold increase in the antiproliferative activity on A431 cells compared to the reference drug gefitinib, while the cyclen derivatives were found the most potent on A549 cells. Further structural optimizations led to the synthesis of compound **27** (Figure 16) by replacing the halogenated aniline moiety at C-4 with 3-chloro-4-((3-fluorobenzyl) oxy)-aniline. Compound **27** was found to effectively deplete intracellular ATP at 8 μM. It displayed a significant increase in the antiproliferative activity on both cell lines (A431 0.95 µM and A549 3.4 µM) compared to gefitinib (2.47 µM and 11.08 µM) and lapatinib (2.66 µM and 3.64 µM). Compound **27** is a potent inhibitor of both EGFR (1.4 nM) and HER2 (2.1 nM) with a nine-fold higher affinity for EGFR^T790M^ (16.5 nM) compared to gefitinib (148.7 nM) (Table S1) [134].

Amrhein et al. synthesized a series of novel macrocyclic quinazoline derivatives as potential EGFR inhibitors with increased specificity toward the common mutant forms of EGFR. Preliminary SAR studies revealed that the length of the macrocycle had an impact on the kinase inhibition profile: shorter linker chains led to increased EGFR^del19^ activity (IC_50_ = 20–236 nM), lower activity on EGFR^L858R^ (IC_50_ = 128–821 nM), and undetectable activity on EGFR^wt^, while longer linker chains led to an even greater increase in the inhibitory activity (EGFRdel19 and EGFRL858R) but with less selectivity toward EGFR^wt^. The most promising compound **28** (Figure 16) displayed an increased selectivity toward EGFR^del19^ kinase (IC_50_ = 119.1 nM) with an undetectable affinity for EGFR^wt^ (IC_50_ ≥ 10 µM) and an excellent selectivity profile kinome-wide. Cytotoxicity assays revealed that compound **28** had no activity on Ba/F3-EGFR^wt^ (IC_50_ ≥ 10 µM) and elevated activity toward Ba/F3-EGFR^L858R^ (IC_50_ = 385.6 nM), Ba/F3-EGFR^L858R/C797^ (IC_50_ = 749.6 nM), Ba/F3-EGFR ^del19^ (IC_50_ = 147.9 nM), and Ba/F3-EGFR ^del19/C797S^ (IC_50_ = 197.5 nM) (Table S1) [135].

### 4.15. Acetyl Glucose-Modified 4-Anilino-quinazoline Derivatives

**Figure 17:**
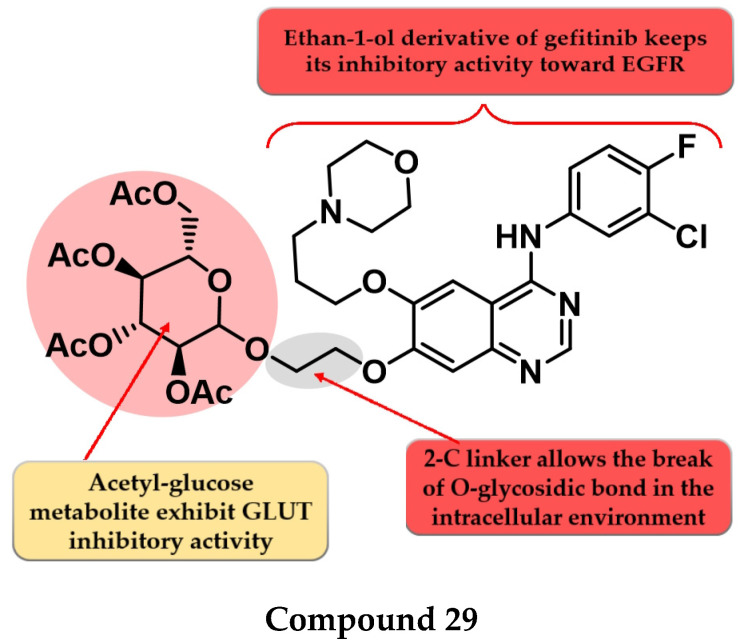
Structure–activity relationship of compound **29** as reported by Yamahana et al. [136].

To enhance the radio-sensitizing effect while maintaining the inhibitory activity toward EGFR, Yamahana et al. synthesized a novel series of acetyl glucose-modified gefitinib derivatives. The compounds displayed antiproliferative activity (IC_50_ = 34.3–39.4 nM) similar to gefitinib (IC_50_ = 31.2 nM) and a significantly higher radio-sensitizing effect. A longer alkyloxy linker (2–4 carbons) between the quinazoline core and the acetyl glucose moiety allows the break of the *O*-glycosidic bond and the generation of an alkyl-7-ol derivative of gefitinib metabolite (which keeps the inhibitory activity toward EGFR). The most active compound **29** (Figure 17) displayed increased EGFR inhibitory activity and cytotoxicity (Table S1) [136].

## 5. Second Generation of EGFR TKIs

The second-generation EGFR TKIs are defined as covalent inhibitors capable of binding to the active site of the EGFR tyrosine kinase and forming an irreversible bond with specific amino acids through an electrophilic warhead called “Michael acceptor” [137,138]. Their development was possible after the discovery through bioinformatics analysis of the Cys797 residue of the EGFR kinase domain in the solvent-accessible region that became the key nucleophilic target for the next generation of covalent inhibitors [138]. Targeting cysteine residues for the development of covalent inhibitors has been extensively studied due to the low prevalence in the proteome and overall better selectivity index [139]. The theoretical advantages of the second-generation inhibitors over the first-generation inhibitors include an increased affinity for the mutant target forms, a more complete blockage of EGFR signaling by inhibiting the heterodimerization partners such as HER2/HER4, and long-term binding that could lead to delay or suppression of the secondary gatekeeper mutation T790M development [137,140].

**Figure 18:**
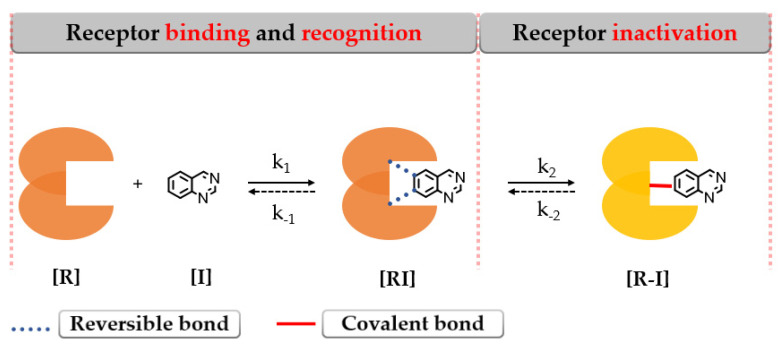
The 2-step formation of a covalent bond between the target [**R**] and inhibitor [**I**] [141].

Covalent binding of the protein leads to enzymatic inactivation maintaining the activity depending on the re-synthesis of the protein [140]. The covalent bond formation follows a two-step interaction with the biological target as depicted in Figure 18.

The first step addresses the formation of an intermediary complex [**RI**] (described by k_1_) like in the case of the first-generation inhibitors by binding to the active site of EGFR and forming weak reversible interactions in a manner that the electrophilic warhead is brought into the proximity of the targeted cysteine residue. The second step is represented by the spontaneous formation of a covalent bond between the electrophilic warhead and the nucleophilic moiety of the inhibitor [**R**–**I**] (described by k_2_) (Figure 18) [141,142,143].

**Figure 19:**
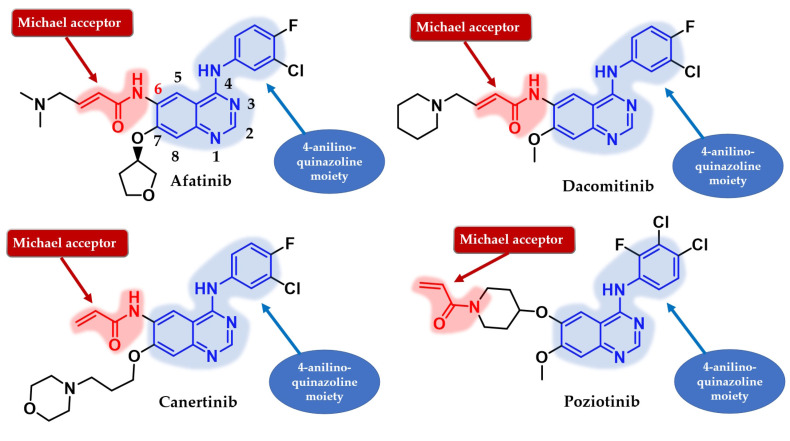
Chemical structure of second-generation EGFR inhibitors quinazoline derivatives approved in therapy or under clinical trial: afatinib, dacomitinib, canertinib, and poziotinib.

Several attempts have been made to develop second-generation EGFR TKIs (Figure 19) [137]. Two covalent 4-anilino-quinazoline inhibitors have been approved for clinical use in the treatment of NSCLC: afatinib in 2013 (BIBW2992, Gilotrif^®^—approved worldwide as first-line monotherapy for adults with locally advanced or metastatic NSCLC with del19 or L858R mutations detected by an FDA-approved test) [144] and dacomitinib in 2018 (PF-002998044, Vizimpro^®^—the same therapeutic indications described for afatinib) [145]. Compared to the first-generation inhibitors, clinical data suggest the utility of second-generation inhibitors such as afatinib as first-line treatment for patients harboring uncommon EGFR mutations (G719S, S768I, and L861Q) [146,147]. Other uncommon mutations, such as exon 20 insertions, can be targeted with second-generation inhibitors. Dacomitinib was found particularly active in glycine insertion at position 770 [148] while a broad-spectrum inhibition of various exon 20 insertions was achieved by the combination of afatinib and cetuximab [149].

The preliminary results of the LUX-Lung 7 clinical trial revealed an increased PFS (47% vs. 41%), higher ORR (70% vs. 54%), and time to treatment failure (TTF) in the afatinib treatment group compared to gefitinib for patients with adenocarcinoma and activating EGFR mutations with minimal OS differences (27.9 vs. 25 months) [150,151]. Better overall clinical usage of second-generation inhibitors compared to first-generation inhibitors is related to their increased potency [151,152] and pan-inhibitory effect over the entire ErbB family of kinases, the overexpression of receptors such as HER2 being associated with tumor resistance in NSCLC patients treated with first-generation inhibitors [153,154]. Even if second-generation inhibitors display a broad-spectrum inhibitory activity toward a large panel of ErbB family kinases, including EGFR^T970M^ mutants [155], the clinical utility for NSCLC patients with developed resistance after treatment with first-generation inhibitors is limited due to increased inhibitory activity toward EGFR^wt^ and significant gastrointestinal and dermatological side effects [156,157].

LUX-Lung 3 and LUX-Lung 6 evaluated the efficiency of the first-line treatment afatinib compared to pemetrexed + cisplatin and gemcitabine + cisplatin. The results indicated a significantly higher ORR in the afatinib group (56% vs. 23% and 67% vs. 23%) and median duration of response (11.1 vs. 5 months and 9.7 vs. 4.3 months), but no improvement for OS compared to chemotherapy in the EGFR-mutation-positive lung adenocarcinoma group [158,159,160].

**Figure 20:**
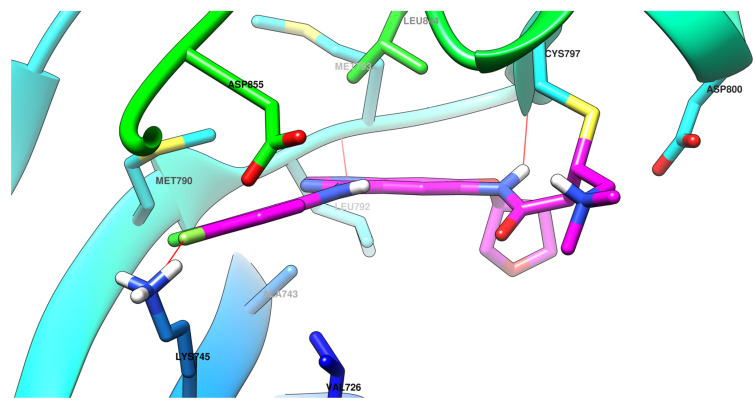
Visualization of afatinib binding to the human EGFRT790M kinase domain co-crystallized in the complexes 4G5P [108] from Protein Data Bank [102] performed using Chimera 1.10.2 (University of California, USA) [103].

The efficiency of a covalent inhibitor is quantitatively evaluated by the second order of rate constant that describes the protein covalent modification k_2_/k_1_ [161]. The ideal covalent inhibitor is characterized by increased k_1_ (nM range), which increases the residence time of the inhibitor near the nucleophilic moiety, and low to moderate reactivity and value of k_2_ (Figure 18) [141,162]. The increased intrinsic affinity of the inhibitor for the target is achieved for EGFR TKIs by following the general principles of drug design for the first-generation EGFR TKIs [28,91]. Most second-generation inhibitors have a 4-anilino-quinazoline base structure for increased affinity (Figure 19) and form similar bonds within the hinge region and the hydrophobic pockets of the active site [108,163,164]. The binding mode of afatinib in the active site of EGFR^T790M^ is presented in Figure 20.

The position and the nature of the electrophilic moiety in the structure of the covalent inhibitor are detrimental to the development of novel clinical candidates [165,166]. Insertion of the electrophilic moiety in the C-6 position of the quinazoline core leads to proximity to the Cys797 residue in the temporary complex [**R**–**I**] and is favorable for the formation of the covalent bond [**RI**] (Figure 18) [141,166,167]. The reactivity of the electrophilic warhead is often a predictor for the off-target effect and side effects [168]. The acrylamide warhead has become extensively used in covalent inhibitor development due to its electrophilic “softness” that has electron conjugation by resonance over the carbonyl and alkenyl moieties [162,169,170,171]. Further modulation of the electronic density and reactivity can be achieved by inserting various substituents at the β-end of the alkenyl moiety that affects the distribution of the electronic cloud [142,171]. The most common modulation is the attachment of an alkaline group to the acrylamide moiety (afatinib and dacomitinib—Figure 19) that elevates the biological activity through intramolecular catalysis of the covalent bond formation [172].

## 6. Novel Second-Generation Quinazoline EGFR TKIs (2017–Present)

### 6.1. α-Chlorofluoro Acetamide Derivatives

**Figure 21:**
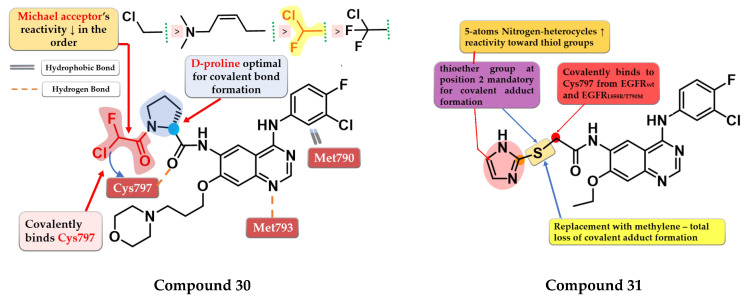
Structure–activity relationship and molecular docking of compound **30** covalently bonded in the human EGFR^L858R/T790M^ kinase domain (PDB ID 5Y25), as reported by Shindo et al. [173]; structure–activity relationship of compound **31**, as reported by Castelli et al. [174].

Shindo et al. synthesized a series of novel quinazoline derivatives bearing an α-chloro-fluor-acetamide covalent binding group, that displayed significantly less reactivity and higher selectivity toward EGFR and mutant forms of EGFR. The presence of a D-proline linker is vital for inhibitory activity by generating the optimal distance between the electrophilic moiety and the reactive thiol group of Cys797. Preliminary SAR studies led to the synthesis of compound **30** (Figure 21) which displayed antiproliferative activity on H1975 cells similar to afatinib (0.19 µM), and less potency toward A431 (7.3 vs. 1.79 µM) and two EGFR-independent cells (HEK293 9.06 vs. 1.78 µM and SW620 10.4 vs. 2.98 µM) (Table S1). The compound displayed a four-fold higher selectivity and lower toxicity with similar excellent potency in growth inhibition in a mouse xenograft model (H1975 cells) compared to the reference drug afatinib. X-ray crystallography data revealed that compound **30** effectively inhibits EGFR^L858R/T790M^ double mutant kinase activity irreversibly by binding the Cys797 residue of the active site with a higher selectivity compared to afatinib due to the shorter length between the electrophilic warhead and the reactive thiol group (3.83 Å vs. 4.29 Å) (Figure 21) [173].

### 6.2. 6-Heteroaryl-thioacetamide-4-anilino-quinazoline Derivatives

Castelli et al. synthesized a novel series of heteroaryl-thioacetamide derivatives as potent covalent inhibitors of EGFR. Preliminary studies revealed that the thioacetamide derivatives displayed a significantly lower reactivity (t_1/2_ > 1440 min) compared to chloroacetamide and acrylamide homologs and a higher selectivity toward the EGFR kinase domain. SAR studies of the synthesized compounds concluded that a pentacyclic nitrogen-bearing heterocycle (imidazole and tetrazole) with the thioether group in position 2 increases the reactivity and the chance of forming the covalent adduct. The covalent adduct formation of the most active compound **31** (Figure 21) was confirmed by both QM/MM simulations and EGFR inhibitory activity on A549 cells after 8 h of washout. Compound **31** displayed a sub-nanomolecular inhibition potency toward EGFR^wt^ kinase (0.62 nM) comparable to the reference drug gefitinib (0.47 nM) and with less potency toward EGFR^L858R/T790M^ after 5 h incubation (≈190 nM) compared to chloroacetamide homolog “8” (structure not provided) in the same conditions (≈4 nM). Compound **31** displayed significant antiproliferative activity on H1975 cells (1.4 µM) compared to the reference drug gefitinib (9.1 µM) (Table S1) [174].

### 6.3. 6-Acrylamide-4-amino-quinazoline Derivatives

**Figure 22:**
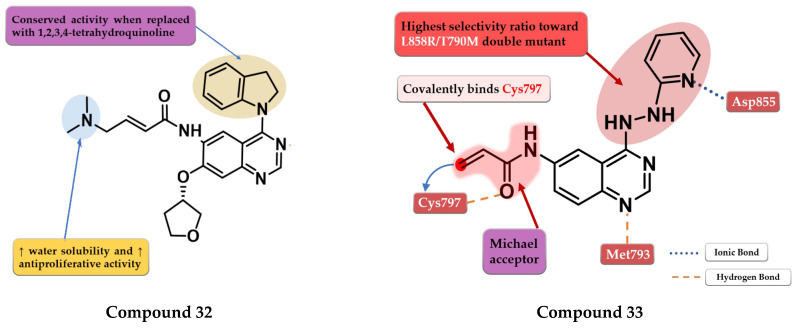
Structure–activity relationship and molecular docking of compound **32** covalently bonded in the active site of EGFR^T790M^, as reported by OuYang et al. [175]; structure–activity relationship and molecular docking of compound **33** covalently bonded in the human EGFR^L858R/T790M^ kinase domain (PDB ID 3IKA) [177], as reported by Pawara et al. [176].

OuYang et al. synthesized a series of novel quinazoline derivatives as potential inhibitors of EGFR kinase. The replacement of the aniline moiety with novel bicyclic amines led to conserved inhibitory activity toward EGFR. Compound **32** (Figure 22) displayed the highest antiproliferative activity on all the tested cells (A549 1.09 µM, MCF-7 1.34 µM, and PC-3 1.23 µM) comparable with that of the reference drug afatinib (0.71 µM, 0.93 µM, and 2.51 µM) (Table S1). The insertion of a tertiary amine at the C-6 position of the quinazoline side chain led to increased activity compared to unsubstituted enamide derivatives due to favorable pharmacokinetics. Compound **32** was identified as a potent inhibitor of EGFR^wt^ kinase (IC_50_ = 5 nM) comparable to the reference drug afatinib (IC_50_ = 5 nM) while exhibiting moderate inhibitory activity toward EGFR^T790M^ kinase (IC_50_ = 26 nM) [175].

Pawara et al. synthesized a series of novel N-(4-substituted-quinazolin-6-yl)-acrylamide derivatives as irreversible EGFR inhibitors by inserting various amino groups containing heterocyclic and homocyclic moieties at the C-4 position of the quinazoline core. All the compounds displayed a significantly higher antiproliferative activity on NCI-H1975 (0.171–0.250 μM) compared to gefitinib (11.71 μM) and similar activity to the reference drug WZ4002 (0.202 μM). Compound **33** (Figure 22) displayed the highest antiproliferative activity on NCI-H1975 cells and a 3.25-fold higher inhibitory activity toward EGFR^L858R/T790M^ compared to EGFR^wt^, with a selectivity pattern like that of WZ4002 (3.15 vs. 2.87) (Table S1). The high selectivity of compound **33** could be correlated with the flexible hydrazine moiety which allows the generation of novel interactions of the pyridine moiety with the DFG motif of the EGFR^L858R/T790M^ [176].

**Figure 23:**
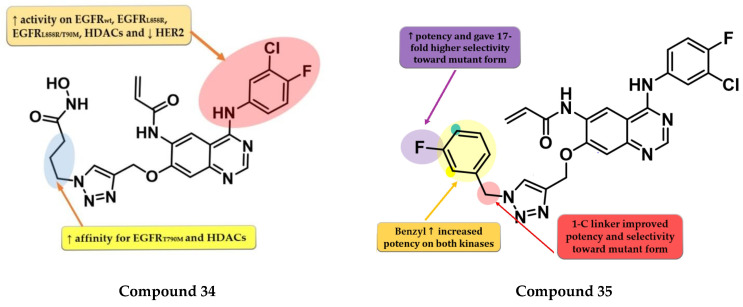
Structure–activity relationship of compound **34**, as reported by Zhao et al. [178]; structure–activity relationship of compound **35**, as reported by Song et al. [179].

Zhao et al. synthesized a series of novel quinazoline derivatives as multitarget ErbB/HDAC inhibitors. Smaller lipophilic substituents on the aniline moiety increased the affinity toward EGFR^T790M/L858R^ compared to bulky ethers. The insertion of a propyl linker between the triazole core and hydroxamic acid moiety led to increased inhibitory activity on EGFR^T790M/L858R^ and HDAC. The most active compound **34** (Figure 23) displayed a higher affinity toward EGFR^T790M/L858R^ (IC_50_ = 1.5 nM) compared to the reference drug afatinib (IC_50_ = 3 nM) and similar affinity for EGFR^wt^ and EGFR^L858R^ (0.35 nM and 1.1 nM). Compound **34** displayed a high selectivity for NCI-H1975 cells with minimal antiproliferative activity on A549, NCI-H838, SK-BR-3, and A431 cells (Table S1). The increased antiproliferative activity on EGFR^T790M^-bearing cells is correlated with the dual mechanism of action of both direct inhibition of EGFR^T790M^-phosphorylation and inhibition of EGFR^T790M^ and Akt intracellular synthesis (hyperacetylation of HSP90 and reduction in its chaperone activity by inhibition of HDACs) [178].

Song et al. synthesized a series of novel quinazoline-triazole hybrids with a higher affinity toward the mutant form EGFR^T790M/L858R^. The substitution in position 1 of the triazole ring with small and hydrophilic substituents (4-hydroxypropyl and morpholino-propyl) showed increased activity but a lower specificity ratio toward EGFR^T790M/L858R^. The replacement of those groups with a benzene ring linked by a one-carbon spacer led to the highest inhibitory activity on all the kinases and the best selectivity ratio. The substitution of the benzyl moiety with small hydrophobic substituents (-F) led to the highest selectivity toward the mutant form. Further modifications led to the conclusion that the *meta*-fluor-substituted compound **35** (Figure 23) had the highest selectivity ratio out of all the synthesized compounds, with a 17-fold higher affinity for EGFR^T790M/L858R^ than EGFR^wt^. The triazole–quinazoline hybrids displayed lower antiproliferative activity (0.5 µM) than afatinib (0.131 µM) on H1975, but significantly higher compared to gefitinib (>10 µM) (Table S1) [179].

### 6.4. Dihydro-6H-[1,4]Oxazino [3,2-g]Quinazoline Acrylamide Derivatives

**Figure 24:**
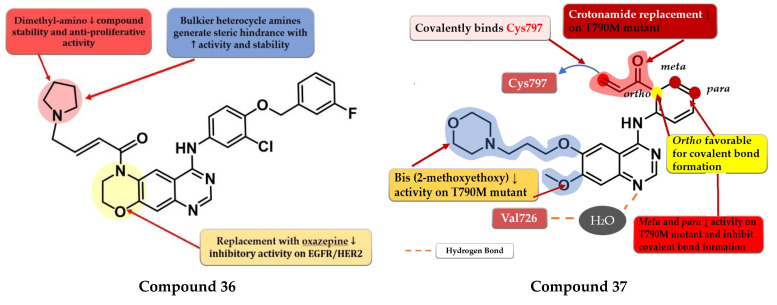
Structure–activity relationship of compound **36**, as reported by Sun et al. [180]; structure–activity relationship and molecular docking of compound **37** covalently bonded in the human EGFR^T790M^ kinase domain (PDB ID 4P5G), as reported by Liu et al. [181].

To overcome the developed tumor resistance for the first- and second-generation EGFR TKIs, Sun et al. synthesized a novel series of tricyclic oxazine fused quinazoline derivatives as dual HER2 and EGFR inhibitors with an irreversible binding mode. The oxazine derivatives were found to be more potent inhibitors of both kinases compared to oxazepine derivatives due to a lack of cell permeability. Dimethyl-amino substitution of the acrylamide moiety led to a flexible side chain and potent dual EGFR/HER2 inhibitors, but low stability due to rapid degradation through hydrolysis and therefore low antiproliferative activity in vivo. Highly potent dual inhibitors were obtained by replacing the dimethyl-amino moiety with bulkier heterocycle amines that led to steric hindrance and more stable compounds. The synthesized compounds displayed higher antiproliferative activity on NCI-H1975 cells harboring EGFR^L858R/T790M^ (IC_50_ = 1.22–4.35 μM) compared to the reference drugs lapatinib, gefitinib, and erlotinib (7.37 µM, >10 µM, and 5.51 µM) (Table S1). The most active compound **36** (Figure 24) displayed remarkable antiproliferative activity on cells harboring both overexpression of EGFR^wt^ or EGFR^L858R/T790M^ and HER2 compared to the reference drug lapatinib and a three-fold more potent inhibition of EGFR in the enzyme assay (7 nM vs. 22 nM) [180].

### 6.5. N-(3-(Quinazolin-4-yl-amino)phenyl)acrylamide Derivatives

Liu et al. synthesized a series of novel quinazoline derivatives as potent inhibitors of EGFR^L858R/T90M^ by incorporating the acrylamide moiety in various positions of the aniline ring. *Ortho*-substitution of the aniline moiety with acrylamide led to the synthesis of the most promising compound **37** (Figure 24). The *meta* or *para* insertion of the acrylamide moiety or its replacement with a crotonamide group led to decreased activity and incapability to generate covalent bonds with Cys797. Compound **37** displayed significant inhibitory activity toward EGFR^L858R/T90M^ (IC_50_ = 4.3 nM), low potency toward EGFR^wt^ (IC_50_ = 104 nM), and a comparable selectivity index with rociletinib (24.4 vs. 25). Molecular docking of compound **37** revealed a shift in the binding position in the active site of EGFR^L858R/T790M^ with the C-6 substituent occupying the hydrophobic pocket I, C-7 substituent trapped in the purine binding area, and the acrylamide-substituted aniline moiety extended in the hydrophilic area capable of generating a covalent bond with Cys797. Compound **37** displayed moderate antiproliferative activity on H1975, A431, and HCC827 (8.7 µM, 13.45 µM, and 0.65 µM) cells and it was less active than the reference drugs gefitinib and rociletinib (0.137 µM, 1.29 µM, and 0.031 µM) (Table S1) [181].

### 6.6. 2,5-Diazabicyclo [2.2.1]Heptane Linked 4-Anilino-quinazoline Acrylates

**Figure 25:**
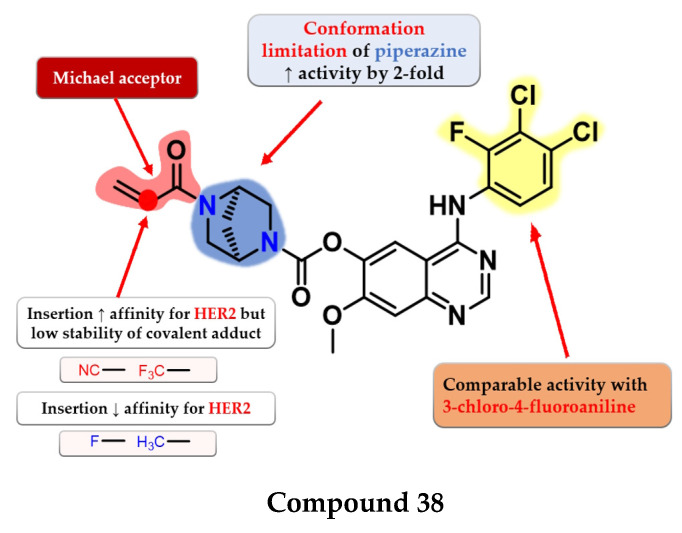
Structure–activity relationship of compound **38**, as reported by Jiao et al. [182].

Jiao et al. synthesized a series of novel 4-anilino-quinazoline as dual covalent inhibitors of EGFR/HER2. The further replacement of the 3-chloro-4-fluoroaniline moiety with various aryl-amine derivatives (polyhalogenated anilines and aromatic heteroaryl amines) failed to improve the inhibitory activity toward EGFR and provided low affinity toward HER2. An increase in the inhibitory activity of HER2, while maintaining the high affinity toward EGFR, was obtained by totally removing the piperazine moiety (which led to low antiproliferative activity due to the low stability of the ester) and the limitation of the conformation of the piperazine by replacing it with 2,5-diazabicyclo-[2.2.1]heptane. The replacement of the Michael acceptor with a 2-fluoro-acrylamide or methyl-acrylamide moieties led to decreased activity on HER2 and conserved activity on EGFR. Further substitution with trifluoromethyl or cyan of the acrylamide moiety led to increased HER2 inhibitory activity but lower antiproliferative activity on all the tested cells due to the low half-life of the covalently bonded warhead. The most promising compound **38** (Figure 25) displayed similar inhibitory activity toward EGFR (IC_50_ = 0.3 nM) and two-fold lower activity on HER2 (IC_50_ = 6.07 nM) compared to the reference drug afatinib (0.27 nM and 3.88 nM) as well as promising antiproliferative activity (Table S1) [182].

## 7. Third Generation of EGFR TKIs

Gatekeeper mutation T790M leads to increased affinity for ATP (lower Michaelis Menten constant—Km) that mediates the resistance to quinazoline derivatives acting as first- and second-generation inhibitors of EGFR [115]. Superior affinity for the T790M form has been achieved by replacing the quinazoline nucleus with a more flexible core (pyrimidine) which led to the development of the third generation of EGFR TKIs [183,184,185,186]. Third-generation EGFR inhibitors are defined as covalent inhibitors with an increased binding affinity for EGFR^T790M^, significantly less affinity for EGFR^wt^, and maintained sub-nanomolecular IC_50_ for common activating mutations (L858R and del19) [187,188,189].

**Figure 26:**
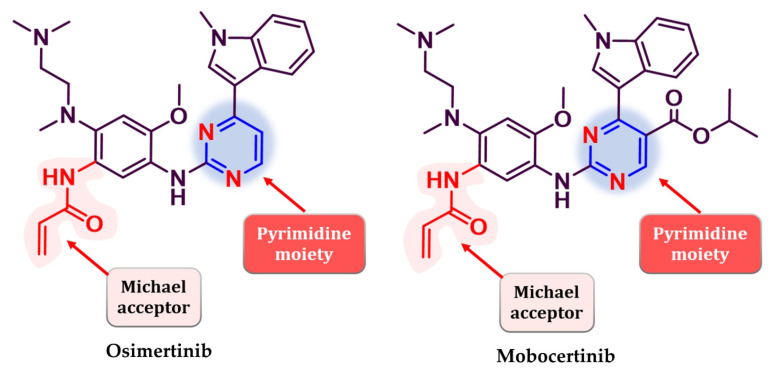
Chemical structure of third-generation EGFR approved in therapy: osimertinib andmobocertinib.

Osimertinib (Figure 26) (AZD9291, Tagrisso^®^) is the first third-generation EGFR TKI approved as first-line therapy for EGFR-positive activating mutations (L858R and del19), NSCLC, and metastatic EGFR^T790M^-positive NSCLC patients detected by an approved FDA test [190]. Its efficiency in patients with a T790M-acquired mutation was established in the FLAURA 3 clinical trial which compared osimertinib with platinum–pemetrexed therapy. Clinical data revealed an increased PFS for osimertinib (10.1 vs. 4.4 months) among all groups (including patients with CNS metastasis) and higher ORR (71% vs. 31%) [191]. Mobocertinib (TAK-788, Exkivity^®^) (Figure 26) is the latest third-generation EGFR inhibitor approved for the treatment of locally advanced or metastatic NSCLC with exon 20 insertion, following an accelerated approval by the FDA in 2021 [192,193,194,195].

Resistance to third-generation inhibitors occurs after 9–13 months of treatment and is mediated by the development of secondary mutations (L858R/C797S) when used as first-line treatment for EGFR-positive NSCLC with common mutations, and tertiary mutations (L858R/T790M/C797S) when used in patients that harbor the T790M mutant after treatment with first and second generation inhibitors by replacing the cysteine covalently bound by the Michael acceptor with a less reactive serine residue [77,196,197,198]. While the L858R/C797S mutant can be overcome by the switch to a first-generation inhibitor that does not rely on a covalent bind to Cys797, of particular interest is the triple L858R/T790M/C797S cis mutant (with the two resistance-conferring mutations on the same allele) which is refractory to EGFR TKI from first generation to third generation [199,200]. Given the definition of third-generation inhibitors, to the best of our knowledge, no quinazoline derivative has been reported as a third-generation inhibitor in the given time (2017–present) even though Section 5 and Section 6 of this work count several molecules active as inhibitors for EGFR^T790M^.

## 8. Fourth Generation of EGFR TKIs

C797S mutation was found to be responsible for 20% of reported cases of resistance to third-generation inhibitors [201,202]. Fourth-generation inhibitors were specifically developed to overcome tumor resistance conferred by triple mutant EGFR^L858R/T790M/C797S^ by binding to other regions of the receptor that are not affected by mutations [203,204]. Cellular signaling is disrupted by the binding of inhibitors into the allosteric pocket (“allosteric inhibitors”) created by the displacement of the regulatory C-helix in the inactive conformation of EGFR kinase with increased affinity for mutant forms and minimal disruption of EGFR^wt^ [205]. Most of the fourth-generation inhibitors displayed good in vitro activity toward EGFR^L858R/T790M/C797S^ kinase but low in vivo effectiveness, leading to the combination with EGFR-targeted antibodies [202,205]. Most fourth-generation inhibitors display minimal to no effect on EGFR^del19/T790M/C797S^ kinase [205]. In a novel approach, inhibitors that bind both to the allosteric pocket and to the ligand-binding region have been developed (ATP-competitive) [206,207,208].

**Figure 27:**
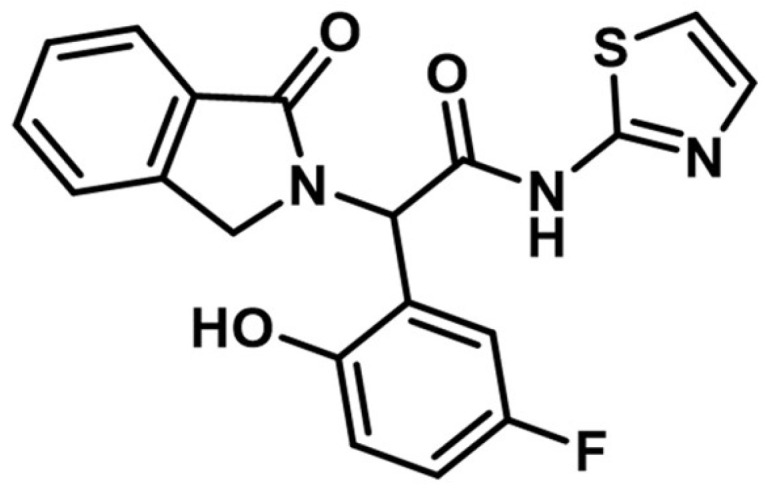
Chemical structure of fourth-generation allosteric EGFR inhibitor EAI045.

The discovery of the fourth-generation inhibitors such as EAI045 (Figure 27) led to a novel approach to EGFR inhibition [205]. Increased affinity for the allosteric pocket is conferred due to the “Y’ shape of the molecule that fits into the region and specifically binds to a Met790 residue of the mutant form [204].

## 9. Novel Fourth-Generation Quinazoline EGFR TKIs (2017–Present)

### 9.1. 2-Aryl-4-amino-quinazoline Derivatives

**Figure 28:**
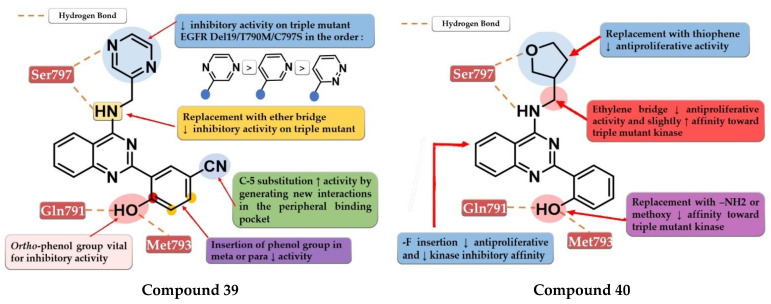
Structure–activity relationship and molecular docking of compound **39** in the EGFR^del19/T790M/C797S^ kinase domain, constructed by homology using EGFR^L858R/T790M^ (PDB ID 3W2R) [209], as reported by Park et al. [208]; structure–activity relationship and molecular docking of compound **40** in the human EGFR^T790M/C797S^ kinase domain (PDB ID 5XGN) [210], as reported by Zhou et al. [207].

Park et al. synthesized a series of novel 2,4-disubstituted quinazoline derivatives as potent inhibitors of the triple EGFR ^d746−750/T790M/C797S^ mutant. In vitro studies revealed a more than 1000-fold selectivity ratio toward the triple mutant compared to EGFR*^wt^* kinase. The presence of 2-hydroxy-phenyl moiety at the C-2 position of the quinazoline core is vital for the inhibitory activity due to multiple H-bonds generated with the ATP-binding pocket. The further substitution of the 2-hydroxy-phenyl moiety in position 5 of the ring (hydroxyl, nitrile, acetyl, methoxy, or methyl-ester) led to a further increase in the inhibitory activity toward the triple mutant, with a conserved selectivity ratio. The terminal groups at the C-5 position of the phenyl moiety occupy the peripheral binding pocket, and increase the inhibitory activity by affecting the functions of the Gly-loop and DFG motif of the kinase domain. Docking simulations of the most active compound **39** (Figure 28) revealed that the presence of an alkaline 6-member heteroaryl moiety and a secondary amine linker is crucial for inhibitory activity by generating strong H-bond interactions with Met793. Compound **39** displayed the highest selectivity ratio toward the triple mutant (IC_50_ = 17.9 nM) compared to EGFR^wt^ (IC_50_ ≥ 50 µM) [208].

Further structural optimizations on compound **39** [208] were realized by Zhou et al. by maintaining the 2,4-substitution pattern on the quinazoline core. The compounds displayed higher antiproliferative activity on A549 cells than on NCI-H460 cells, less potency toward H1975, with more than 10-fold less activity overall compared to the reference drug AZD9291 on all three tested cell lines. The replacement of the phenol group with amino or methoxy groups led to decreased antiproliferative activity and inhibitory activity toward EGFR^L858R/T790M^. The substitution of the C-6 positions of the quinazoline core with fluorine led to decreased activity. Further modulation of the C-4 substituent of the quinazoline core led to the identification of compound **40** (Figure 28), synthesized by the replacement of the lateral pyridine ring of the lead compound with a tetrahydrofuran-3-yl moiety. The replacement of the methyl-amino bridge with longer linkers led to decreased activity. Compound **40** had a 10-fold higher selectivity toward EGFR^L8585R/T790M^ (compared to EGFR^wt^—740 nM and >10^5^ nM) than the reference drug afatinib (10 nM and 6 nM). Compound **40** displayed the highest antiproliferative activity on all the tested cells (A549, NCI-H460, and H1975) and 91% of EGFR^Del19/T790M/C797S^ kinase at 10 µM [207].

### 9.2. 6-Methoxy-7-[(1-methylpiperidin-4-yl) methoxy]quinazolin-4-aniline Derivatives

**Figure 29:**
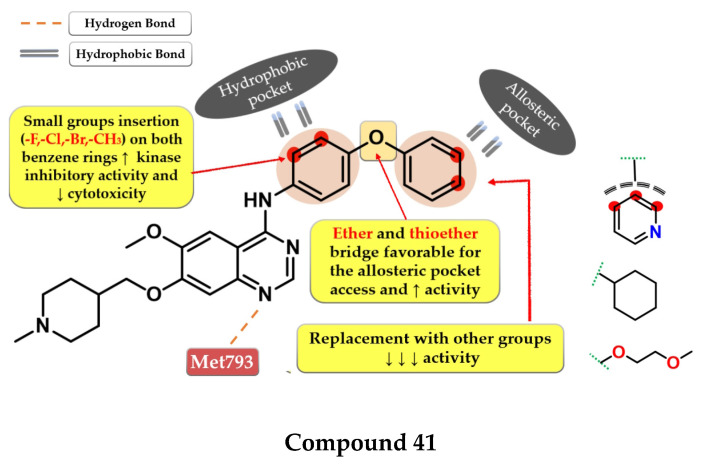
Structure–activity relationship and molecular docking of compound **41** in the molecular model of EGFR^del19/T790M/C797S^ATP-binding domain, build using Maestro (PDB ID 1XKK used as a template) [101], as reported by Dou et al. [211].

Dou et al. developed a series of 4-anilino-quinazoline derivatives as potential fourth-generation EGFR^C797S^ kinase inhibitors. The structural optimization by inserting various groups in the *para*-position of the 4-aniline moiety in the structure of the reference drug vandetanib (Figure 1), a moderate inhibitor of EGFR^L858R/T790M/C797S^ kinase (IC_50_ = 369 nM), led to the synthesis of a series of novel potent compounds capable of spanning both the ATP-binding pocket and the allosteric site. The best results were obtained by inserting a highly lipophilic group linked by a flexible linker (ether or thioether bridge) capable of generating a suitable binding posture and torsion angle for a strong interaction with the allosteric site. The most active compound **41** (Figure 29) displayed an increased inhibitory activity toward EGFR^L858R/T790M/C797S^ kinase (IC_50_ = 0.128 µM) with good selectivity and cytotoxic activity on BaF3-EGFR^L858R/T790M/C797S^ (0.75 µM) and BaF3-EGFR^19del/T790M/C797S^ (0.09 µM) cells, comparable with brigatinib (0.56 µM and 0.17 µM) (Table S1). The replacement of the lateral phenyl group with other groups (pyridine and cyclohexyl) led to a drastically decreased in vitro activity, while the further substitution with various small lipophilic substituents (-F, -Cl, and -Br) and EDGs (-CH_3_) led to increased kinase inhibitory activity but less potent cytotoxic activity due to poor pharmacokinetics [211].

**Figure 30:**
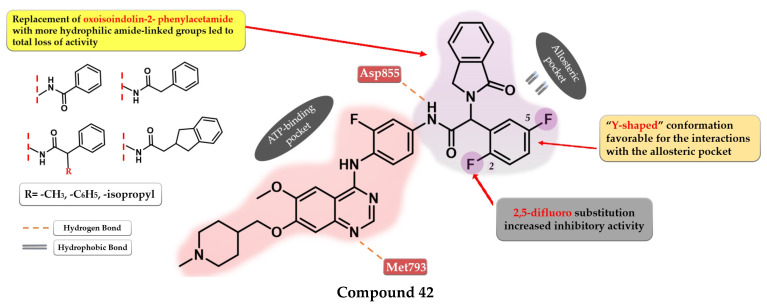
Structure–activity relationship and molecular docking of compound **42** in the human EGFR^del19/T790M/C797S^ kinase domain (PDB code 5D41), as reported by Li et al. [206].

Li et al. synthesized a series of novel reversible EGFR^L858R/T790M/C797S^ quinazoline derivatives by merging the structural design of vandetanib (Figure 1) and EAI045 (Figure 26). Increased inhibitory activity was achieved by inserting the oxoisoindolin-2-phenylacetamide residue in the *para*-position of the 4-amino-phenyl moiety by generating a “Y-shaped” conformation capable of occupying the allosteric pocket. Moving the substituent on the aniline moiety in *meta*-position led to a four-fold decrease in the inhibitory activity, while the replacement with other more hydrophilic amide-linked groups led to a total loss of activity. The 2,5-difluoro substitution of the lateral phenyl ring led to increased binding affinity and the synthesis of compound **42** (Figure 30), the most active of the series. Compound **42** displayed a significant inhibitory activity toward EGFR^L858R/T790M/C797S^ (IC_50_ = 2.2 nM) and an increased cytotoxic effect on BaF3-EGFR^L858R/T790M/C797S^ (0.64 µM), comparable to the reference drug brigatinib (0.42 µM) and higher than that of afatinib (3.93 µM) (Table S1) [206].

## 10. Conclusions

The identification of the quinazoline core as a valuable scaffold for EGFR inhibition led to the development of several TKIs with tremendous clinical utility in various types of cancer (especially NSCLC). The identification of the key structural features of an ideal EGFR TKI (increased potency toward mutant forms of EGFR, minimal affinity for EGFR^wt^, and low off-target effect) has been a hot topic for many research groups for some time. This work provides a collection of general drug design rules for the development of novel quinazoline derivatives as potential EGFR inhibitors based on new quinazoline derivatives synthesized in the last 6 years.

The substitution pattern on the quinazoline core plays a crucial role in the development of novel potent EGFR TKIs. Along with the already-established structure design principles (aniline moiety in position 4, electron-donating groups in positions 6 and 7, Michael acceptor inserted from the C-6 substituents) for the development of EGFR TKIs, this work outlines novel approaches to increasing the inhibition potential even further while maintaining a high selectivity index. Newly synthesized first-generation quinazoline inhibitors have lower IC_50_ for common mutation forms of EGFR (del19 and L858R) and increased affinity for secondary mutations (T790M) compared to the inhibitors already approved in therapy. This was achieved by structural modulations such as the replacement of various substituents in order to generate novel bonds with the target, and the development of multi-target inhibitors with favorable pharmacokinetics. A better understanding of the covalent bond formation for the second-generation inhibitors led to the discovery of novel Michael acceptors (α-chlorofluoro-acetamides, thioacetamides, and novel substitution patterns on the already-established acrylamide moiety), used for the development of novel EGFR quinazoline TKIs with improved balance between reactivity and affinity for the target.

A breakthrough is the discovery of the fourth-generation inhibitors, bearing a quinazoline core, as allosteric inhibitors of the triple mutant L858R/T790M/C797S highly resistant to the first three generations of inhibitors. This was achieved by the further substitution of the aniline moiety with various bulky substituents capable of occupying the allosteric pocket, or a non-canonical 2,4-substitution profile capable of generating a new binding mode into the active site of the kinase.

For our research group, this work will provide a solid base for the development of a series of original articles focused on the synthesis of novel quinazoline derivatives as potential EGFR TKIs.

## Data Availability

Data sharing not applicable.

## Supplementary Materials

The following supporting information can be downloaded at: https://www.mdpi.com/article/10.3390/ph16040534/s1. Figure S1. Bibliometric graphic of the reported series of quinazoline derivatives as EGFR TKIs found in the Scopus database (blue) and selected series for the discussion of SAR (red) in the period 2017–present; Table S1: In vitro efficiency of novel quinazoline derivatives (2017–present) as potential EGFR inhibitors.
