# Supplementary material for: Structure–Activity Relationship Studies Based on Quinazoline Derivatives as EGFR Kinase Inhibitors (2017–Present)
Source: Pharmaceuticals (Basel). 2023 Apr 3;16(4):534. doi: 10.3390/ph16040534 (PMC10141396; doi:10.3390/ph16040534)
Supplement: Supplementary file 1 [file pharmaceuticals-16-00534-s001.zip › pharmaceuticals-2302817-supplementary.pdf]

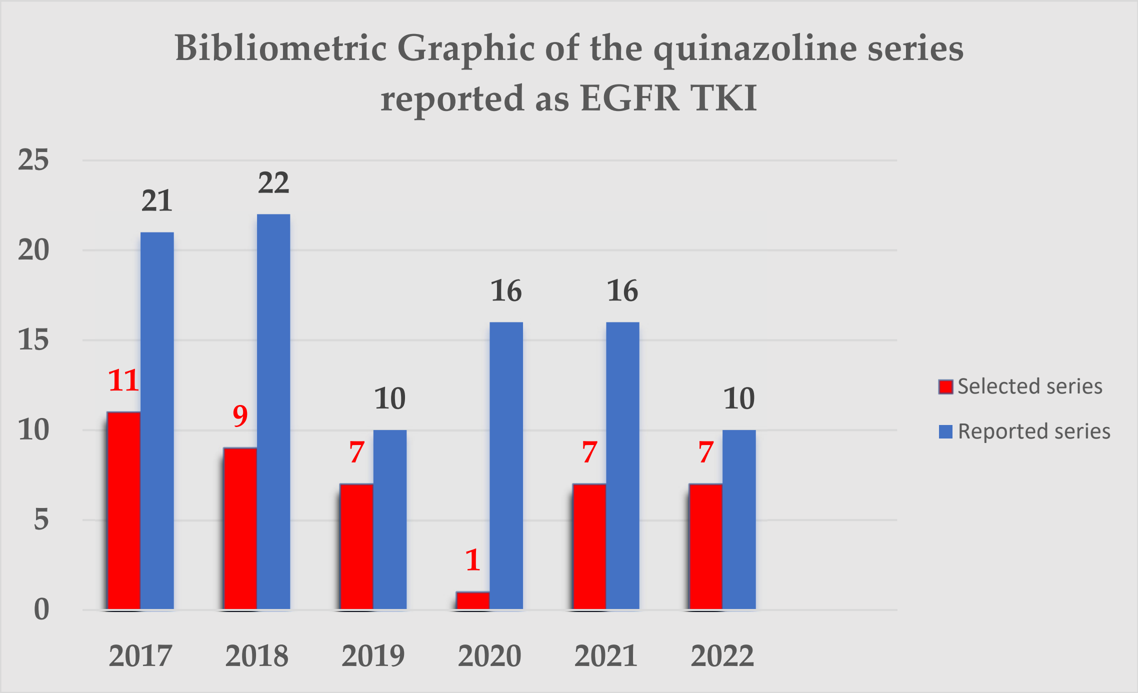

Figure S1. Bibliometric graphic of the reported series of quinazoline derivatives as EGFR TKIs found in the Scopus Database (Blue) and selected series for the discussion of SAR (red) in the period 2017-present

Table S1. *In vitro* efficiency of novel quinazoline derivatives (2017-present) as potential EGFR inhibitors.

Refference	Compound	Kinase assay	Cytotoxicity assay
		Type of kinase (IC_{50} ± SD, nM)	Cell line (IC_{50} ± SD, μM)
Chen et al. [104]	Compound 1	$EGFR^{wt}$ (20.72 ± 6.41)	A431 (1.35 ± 0.32); A549 (21.17 ± 0.47); NCI-H1975 (12.70 ± 2.98); SW480 (12.50 ± 0.28);
	Gefitinib	$EGFR^{wt}$ (3.22 ± 1.48)	A431 (4.45 ± 0.25); A549 (8.83 ± 3.80); NCI-H1975 (5.53 ± 0.30); SW480 (6.08 ± 0.32);
	Lapatinib	$EGFR^{wt}$ (27.06 ± 3.77)	A431 (4.80 ± 0.71); A549 (14.90 ± 1.21); NCI-H1975 (9.08 ± 5.82); SW480 (12.58 ± 1.35);
Zhang et al. [105]	Compound 2	$EGFR^{wt}$(7.0 ± 1.4) $EGFR^{L858R}$(322.5 ± 8.7) $EGFR^{L858R/T790M}$ (>2000) $EGFR^{T790M}$ (9.3 ± 0.9)	A431 (1.31 ± 0.55); A549 (1.65 ± 0.29); NCI-H1975 (4.87 ± 0.68); HCC827 (0.37 ± 0.06) SW480 (3.27 ± 1.25);
	Gefitinib	$EGFR^{wt}$(3.2 ± 1.5) $EGFR^{L858R}$(1.5 ± 0.5) $EGFR^{L858R/T790M}$ (>2000) $EGFR^{T790M}$ (127.9 ± 5.5)	A431 (4.45 ± 0.25); A549 (21.17 ± 0.47); NCI-H1975 (12.70 ± 2.98); HCC827 (1.60 ± 0.30*10^3) SW480 (12.50 ± 0.28);
Tu et al. [106]	Compound 3	EGFR(56)	A549(1.32 ± 0.38); HepG2 (0.07 ± 0.61); MCF-7 (0.91 ± 0.29); PC-3 (0.91 ± 0.29);
	Afatinib	EGFR(1.6)	A549(1.40 ± 0.83); HepG2 (1.40 ± 0.83); MCF-7 (2.63 ±1.06); PC-3 (2.63 ± 1.06);
Wang et al.	Compound 4	$EGFR^{wt}$(6.3);	A549(5.9 ± 0.25); HepG2 (4.63 ± 0.36); MCF-7 (2.37 ±

[107]		EGFR$^{L858R/T790M}$ (8.4)	0.34); H1975 (1.72 ± 0.85);
	Afatinib	EGFR^{wt}(4); EGFR$^{L858R/T790M}$ (3.8)	A549(1.33 ± 0.09); HepG2 (1.40 ± 0.08); MCF-7 (2.63 ± 0.16); H1975 (0.49 ± 0.08);
Zhang et al. [109]	**Compound 5**	EGFR(1), VEGFR2(79)	HT-29(1.76); MCF-7(7.28); H460(26);
	Vandetanib	EGFR(11), VEGFR2(15)	HT-29(18.95); MCF-7(11.83); H460(37.10);
Sun et al. [110]	**Compound 6**	EGFR(10);VEGFR2(80);	HCT116 (8.35); MCF-7(15.66); B16(5.57);
	Sorafenib	EGFR(20);VEGFR2(80);	HCT116 (10.55); MCF-7(17.87); B16(9.29);
Hamad et al. [111]	**Compound 7**	EGFR(60.1); NF-κB (300)	MDA-MB-231(0.9)[a]
	Gefitinib	EGFR(9.7); NF-κB (4000)	MDA-MB-231 (14.2)[a]
Gan et al. [112]	**Compound 8**	EGFR^{wt}(0.8);EGFR$^{L858R/T790M}$ (2.7);	H1975(1.59 ± 0.35); A549(0.27 ± 0.04); HeLa(2.68 ± 0.17); MCF-7(1.26 ± 0.24);
	Afatinib	EGFR^{wt}(0.6);EGFR$^{L858R/T790M}$ (3.5);	H1975(0.49 ± 0.08); A549(1.33 ± 0.09); HeLa(2.63 ± 0.16); MCF-7(1.40± 0.08);
Hou et al. [113]	**Compound 9**	EGFR^{wt}(5); EGFR^{L858R}(2.7); EGFR$^{d746-750}$(1.3);	A431(2.352); H1975(5.34); Ba/F3-EGFR^{L858R}(0.182); Ba/F3 EGFR$^{Del\ E746_A750}$(0.178);HCC82 (0.098); NCI-H1975(5.34);
	Geftinib	EGFR^{wt}(0.5);	A431(0.768); H1975(5.34):
Zhang el al. [114]	**Compound 10**	EGFR^{wt}(93.2 ± 8.0); EGFR^{T790M} (93.2 ± 8.0); SI (2.72)[b]	A549(6.10 ± 0.69); A431(13.86 ± 0.22); H1977(1.22 ± 0.11);
	Geftinib	EGFR^{wt}(17.1 ± 4.2); EGFR^{T790M} (17.1 ± 4.2) SI (0.05)[b]	A549(1.22 ± 0.11); A431(1.22 ± 0.11); H1977(11.29 ± 0.53);
	Erlotinib	EGFR^{wt}(15.3 ± 5.6); EGFR^{T790M} (362.8 ± 64.2) SI (0.04)[b]	A549(11.29 ± 0.53); A431(11.64 ± 0.77); H1977(12.83 ± 0.96);
	Osimertinib	EGFR^{wt}(362.8 ± 64.2); EGFR^{T790M} (13.4 ± 2.5) SI (4.6)[b]	A549(8.36 ± 0.75); A431(5.88 ± 0.46); H1977(0.95 ± 0.03);
Tang et al. [116]	**Compound 11**	EGFR^{wt}(0.72 ± 0.11); EGFR^{T790M} (0.67 ± 0.08); HER2(75.1 ± 8.5); HER4(2.0 ± 0.0);	Good antiproliferative activitiy on A431, NCI-H1975, HCC827, A549, NCI-1650, SK-BR-3, BT-474, SKOV3, T47D, MCF-7, NIH3T3, SW620 cells (exact values not provided)
	Afatinib	EGFR^{wt}(8.2 ± 1.2); EGFR^{T790M} (3.7 ± 1.2); HER2(12.5 ± 0.9); HER4(7.2 ± 2.5);	Good antiproliferative activitiy on A431, NCI-H1975, HCC827, A549, NCI-1650, SK-BR-3, BT-474, SKOV3, T47D, MCF-7, NIH3T3, SW620 cells (exact values not provided)
Zou et al. [117]	**Compound 12**	-	A431(3.4); A439 (>50)
	Erlotinib	-	A431(3.0); A439 (>50)
Zhang et al.	**Compound 13**	EGFR^{wt}(5.06 ± 1.92);	SW480(5.58 ± 1.43); A549(7.35 ± 1.42);

[118]			NCI-H1975(3.01 ± 1.07); A431(3.64 ± 0.51);
	Gefitinib	$EGFR^{wt}$(8.2 ± 1.2);	SW480(12.50 ± 0.28); A549(21.17 ± 0.47); NCI-H1975(12.70 ± 2.98); A431(4.45 ± 0.25);
	Lapatinib	$EGFR^{wt}$(27.06 ± 3.77);	SW480(12.58 ± 1.35); A549(14.90 ± 1.21); NCI-H1975(9.08 ± 5.82); A431(4.80 ± 0.71);
Ding et al. [119]	**Compound 14**	EGFR (0.12 ± 0.03); HER-2(174.9 ± 19.6); HDAC1 (0.72 ± 0.11); HDAC6 (3.2 ± 0.5);	A549 (0.63 ± 0.12); A431 (0.49 ± 0.06); BT-474 (3.88 ± 0.06); SK-BR-3 (0.69 ± 0.03); NCI-H1975(8.05 ± 1.15);
	Lapatinib	EGFR (23.9 ± 1.4);	A549 (1.74 ± 0.28); A431 (0.15 ± 0.01); BT-474 (0.10 ± 0.02); SK-BR-3 (0.06 ± 0.01); NCI-H1975(7.25 ± 0.38);
	Vorinostat	HDAC1 (10.8 ± 0.7); HER2 (10.8 ± 1.4);	A549 (2.57 ± 0.37); A431 (2.29 ± 0.04); BT-474 (2.67 ± 0.38); SK-BR-3 (2.58 ± 0.13); NCI-H1975(1.90 ± 0.09);
Wei et al. [120]	**Compound 15**	EGFR(5.90); VEGFR-2(36.78);	[c]A549(14.87, 72.99[d], 80.31[e]); H446(16.76, 76.34[d], 79.03[e]);
	Vandetanib	EGFR(19.76); VEGFR-2(33.26);	[c]A549(21.06, 67.26[d], 81.44[e]); H446(25.58, 77.67[d], 82.02[e]);
Cheng et al. [121]	**Compound 16**	EGFR(120)	A549 (1.59±0.81, 1.09±0.88[d]); HT-29(2.46±1.56, 1.35±0.91[d]);
	Lapatinib	EGFR(11)	A549 (11.30±2.34, 13.26±3.66[d]); HT-29(6.81±1.24, 8.85±1.05[d]);
Elkamhawy et al. [124]	**Compound 17**	EGFR(1.8) HER2(87.8)	-
	Staurosporine	EGFR(88.1) HER2(35.5)	-
	Lapatinib	EGFR(10) HER2(9)	-
Quin et al. [123]	**Compound 18**	EGFR(10.29)	A549(9.95); NCI-H157 (11.66); T293(>100); WI-38(90.55);
	Gefitinib	EGFR(10.41)	-
	Erlotinib	EGFR(11.65)	A549(7.26); NCI-H157 (6.88); T293(65.56); WI-38(89.38);
Zheng et al. [125]	**Compound 19**	EGFR(3.2±0.2)	HepG2 (8.3 ± 0.6)
	ZM447439	EGFR(110)	HepG2 (1.4 ± 0.2)
Ismail et al. [126]	**Compound 20**	EGFR(37 ± 2)	HepG2(12.00 ± 0.70); MCF-7(3.00 ± 0.10);
	Erlotinib	EGFR(73 ± 5)	HepG2(25 ± 1.50); MCF-7(20 ± 0.93);
Ahmed et al. [127]	**Compound 21**	EGFR(46.90 ± 1.02); HER2(37.64 ± 1.89);	AU-565(1.54 ± 0.08); MDA-MB-231 (2.67 ± 0.16); MCF10A (35.31 ± 1.83);
	Lapatinib	EGFR(53.10 ± 1.2); HER2(56.20 ± 2.99);	AU-565(0.483 ± 0.03); MDA-MB-231 (9.29 ± 0.56); MCF10A (39.57 ± 2.05);

Mphahlele et al. [128]	**Compound 22**	EGFR(40.7 ± 0.31)	A549(89.16); Caco-2(6.45); C3A(12.20); MCF-7(39.07); HeLa(25.51);
	Gefitinib	EGFR(38.9 ± 0.89)	A549(51.29); Caco-2(27.91); C3A(5.01); MCF-7(30.74); HeLa(98.80);
Ding et al. [129]	**Compound 23**	EGFR(2.4);PI3Kα(317) PI3Kβ(9412); PI3Kγδ (3560); PI3Kδ (8672);	A549(8.23 ± 0.34); BT549(1.02 ± 0.08); HCT-116(5.60 ± 0.24); MCF-7(5.59 ± 0.21); SK-HEP-1(6.10 ± 0.26); SNU638(4.10 ± 0.13);
	Gefitinib	EGFR(2.3);	A549(8.27 ± 0.42); BT549(6.56 ± 0.35); HCT-116(5.98 ± 0.72); MCF-7(26.7 ± 1.02); SK-HEP-1(10.1 ± 0.32); SNU638(7.56 ± 0.24);
	Dactolisib	PI3Kα(16.4) PI3Kβ(35.9); PI3Kγδ (23.6); PI3Kδ (78.4);	A549(0.62 ± 0.07); BT549(0.74 ± 0.08); HCT-116(0.84 ± 0.12); MCF-7(1.33 ± 0.14); SK-HEP-1(1.82 ± 0.23); SNU638(1.24 ± 0.13);
Zhang et al. [130]	**Compound 24**	$EGFR^{wt}$(27.0 ± 6.8); $EGFR^{T790M}$ (9.2 ± 2.1);	A549(6.54 ± 0.5); A431(4.04 ± 0.34); H1975(1.94 ± 0.14);
	Gefitinib	$EGFR^{wt}$(17.1 ± 4.2); $EGFR^{T790M}$ (378.4 ± 56.8);	A549(15.59 ± 1.03); A431(8.37 ± 0.46); H1975(10.78 ± 0.45);
	Osimertinib	$EGFR^{wt}$(58.2 ± 12.6); $EGFR^{T790M}$ (8.1 ± 2.2);	A549(-); A431(5.32 ± 0.43); H1975(0.98 ± 0.01);
Chang et al. [132]	**Compound 25**	EGFR(3.62)	HepG2(4.61); A549(9.50); DU145(6.79); MCF-7(9.80); SH-SY5Y(7.77);
	Gefitinib	EGFR(3.62)	HepG2(29.79); A549(12.08); DU145(8.63); MCF-7(12.05); SH-SY5Y(18.21);
Wang et al. [133]	**Compound 26**	-	A431(1.27 ± 0.95); A549(1.67 ± 0.38); Hela(3.77 ± 0.63); HL-60(3.11 ± 0.01); SMMC-7721(5.37 ± 0.02); BGC823(1.66 ± 0.38); SK-OV-3(5.26 ± 1.30); HepG2(3.98 ± 0.88);
	Gefitinib	-	A431(12.93 ± 4.54); A549(13.75 ± 5.73); Hela(17.92 ± 1.50); HL-60(17.72 ± 1.76); SMMC-7721(23.27 ± 0.66); BGC823(>10); SK-OV-3(12.31 ± 0.33); HepG2(>10);
Ju et al. [134]	**Compound 27**	$EGFR^{wt}$(1.4 ± 0.2); HER2(2.1 ± 0.6); $EGFR^{T790M}$(16.5 ± 2.3);	A431(0.958 ± 0.034); A549(3.4 ± 0.4);
	Gefitinib	$EGFR^{wt}$(5.8 ± 0.2); HER2(-); $EGFR^{T790M}$(148.7 ± 6.3);	A431(2.47 ± 0.07); A549(11.08 ± 2.29);
	Lapatinib	$EGFR^{wt}$(10.85 ± 0.11); HER2(32.7 ± 1.4); $EGFR^{T790M}$(>2500);	A431(2.66 ± 0.27); A549(3.64 ± 0.77);
Amrhein et	**Compound 28**	$EGFR^{wt}$(>10^4);	Ba/F3-$EGFR^{wt}$(>10^4);

al. [135]		EGFR^{del19}(119.1);<br>EGFR^{L858R}(820);<br>EGFR$^{L858R/T790M}$(>10^{4});	Ba/F3-EGFR^{del19}(0.197);Ba/F3-EGFR$^{del19/C797S}$(0.147);<br>Ba/F3-EGFR^{L858R}(0.385);Ba/F3-EGFR$^{L858R/C797S}$(0.749);<br>Ba/F3-EGFR$^{L858R/T790M}$(>10^{4});
	Gefitinib	EGFR^{wt}(>100);<br>EGFR^{del19}(0.9);<br>EGFR^{L858R}(3.8);<br>EGFR$^{L858R/T790M}$(>100);	Ba/F3-EGFR^{wt}(0.053);<br>Ba/F3-EGFR^{del19}(0.01);Ba/F3-EGFR$^{del19/C797S}$(0.012);<br>Ba/F3-EGFR^{L858R}(0.021);Ba/F3-EGFR$^{L858R/C797S}$(0.054);<br>Ba/F3-EGFR$^{L858R/T790M}$(>10^{4});
	Osimertinib	EGFR^{wt}(34.5);<br>EGFR^{del19}(0.5);<br>EGFR^{L858R}(1.8);<br>EGFR$^{L858R/T790M}$(0.8);	Ba/F3-EGFR^{wt}(0.053);<br>Ba/F3-EGFR^{del19}(0.005);Ba/F3-EGFR$^{del19/C797S}$(1.32);<br>Ba/F3-EGFR^{L858R}(0.002);Ba/F3-EGFR$^{L858R/C797S}$(1.475);<br>Ba/F3-EGFR$^{L858R/T790M}$(0.027);
Yamahana et al. [136]	**Compound 29**	-	A431(31.2);
	Gefitinib	-	A431(39.4);
Shindo et al. [173]	**Compound 30**	-	PC9(0.00042); H1975(0.19); A431(7.30);<br>HEK293(9.06); SW620(10.4);
	Afatinib	-	PC9(0.00042); H1975(0.19); A431(1.79);<br>HEK293(1.78); SW620(2.98);
Castelli et al. [174]	**Compound 31**	EGFR^{wt}(0.62 ± 0.08);	A549(0.027 ± 0.035); H1975(1.4 ± 0.10);
	Gefitinib	EGFR^{wt}(0.47 ± 0.05);	A549(0.060 ± 0.025); H1975(9.1 ± 1.1);
OuYang et al. [175]	**Compound 32**	EGFR^{wt}(5);<br>EGFR^{T790M}(26);	A549(1.09 ± 0.04); MCF-7(1.34 ± 0.13); PC-3(1.23 ± 0.09);
	Afatinib	EGFR^{wt}(5);<br>EGFR^{T790M}(7);	A549(0.71 ± 0.05); MCF-7(0.93 ± 0.09); PC-3(2.51 ± 0.18);
Pawara et al. [176]	**Compound 33**	EGFR^{wt}(20.2 ± 1.2);<br>EGFR$^{L858R/T790M}$(6.2 ± 0.6);	NCI-H1975(0.171 ± 0.019); A549(0.540 ± 0.022);<br>HepG2(1.010 ± 0.29); SI[f](3.15);
	Gefitinib	-	NCI-H1975(11.71 ± 0.22); A549(9.25 ± 0.18);<br>HepG2(51.74 ± 0.27); SI[f](0.78);
	WZN4002	EGFR^{wt}(26.2 ± 1);<br>EGFR$^{L858R/T790M}$(8 ± 0.8);	NCI-H1975(0.202 ± 0.015); A549(0.580 ± 0.035);<br>HepG2(1.200 ± 0.015); SI[f](2.87);
Zhao et al. [178]	**Compound 34**	EGFR^{wt}(0.35);<br>EGFR^{L858R}(1.1);<br>EGFR$^{L858R/T790M}$(1.5);<br>HER2(5.7);<br>HDAC1(75);<br>HDAC2(12);	A549(>50); NCI-H838(>50); SK-BR-3(30.80 ± 7.18);<br>A431(20.65 ± 5.37); NCI-H1975(1.82 ± 0.50);HL-7702(>25); FHC(>25);
	Afatinib	EGFR^{wt}(0.32);<br>EGFR^{L858R}(0.88);<br>EGFR$^{L858R/T790M}$(3);<br>HER2(6.2);	A549(1.90 ± 0.50); NCI-H838(9.92 ± 3.22);<br>SK-BR-3(5.56 ± 1.69); A431(0.59 ± 0.27);<br>NCI-H1975(0.74 ± 0.28);HL-7702(1.62 ± 0.37);<br>FHC(1.55 ± 0.70);
	Osimertinib	EGFR$^{L858R/T790M}$(0.43);	-
	Vorinostat	HDAC1(16);	A549(4.25 ± 0.53); NCI-H838(5.18 ± 2.26);

		HDAC2(13);	SK-BR-3(2.13 ± 1.10); A431(1.47 ± 0.42); NCI-H1975(2.04 ± 0.31);HL-7702(5.45 ± 2.65); FHC(2.59 ± 1.10);
Song et al. [179]	**Compound 35**	$EGFR^{wt}$(45); $EGFR^{L858R/T790M}$(6); HER2(212);	H1975(0.505); PC9(0.0009);A549(5.501); SKBR3 (0.115);
	Gefitinib	$EGFR^{wt}$(12); $EGFR^{L858R/T790M}$(460); HER2(151);	H1975(>10); PC9(0.061);A549(>10); SKBR3 (2.518);
	Afatinib	$EGFR^{wt}$(9); $EGFR^{L858R/T790M}$(14); HER2(37);	H1975(0.131); PC9(0.0001);A549(3.766); SKBR3 (0.0012);
Sun et al. [180]	**Compound 36**	EGFR(7±0.9); HER2(4±0.5);	N87(0.048 ± 0.006); H1975(1.67 ± 0.29); A431(0.633 ± 0.058); BT474(0.060 ± 0.007); Calu-3(0.177 ± 0.062);
	Gefitinib	EGFR(1±0.8); HER2(281±6.1);	N87(1.00 ± 0.1); H1975(>10); A431(0.550 ± 0.063); BT474(0.365 ± 0.044); Calu-3(0.985 ± 0.021);
	Erlotinib	EGFR(1±0.4); HER2(369±9.1);	N87(>10); H1975(5.51 ± 0.7); A431(0.750 ± 0.083); BT474(>10); Calu-3(0.926 ± 0.055);
	Lapatinib	EGFR(22±3.5); HER2(13±4.1);	N87(0.053 ± 0.004); H1975(7.37 ± 0.75); A431(3.59 ± 0.59); BT474(0.037 ± 0.008); Calu-3(0.216 ± 0.035);
Liu et al. [181]	**Compound 37**	$EGFR^{wt}$(105); $EGFR^{L858R/T790M}$(4.3); SI(24.4)[g]	H1975(8.70); A431(13.45); HCC827(0.65); A549(32.42); HBE(34.04);
	Gefitinib	$EGFR^{wt}$(15.5); $EGFR^{L858R/T790M}$(832.3); SI(0.019)[g]	H1975(10.89); A431(3.308); HCC827(0.006); A549(10.07); HBE(23.8);
	Rociletinib	$EGFR^{wt}$(500); $EGFR^{L858R/T790M}$(20); SI(25)[g]	H1975(0.137); A431(1.29); HCC827(0.031); A549(6.5); HBE(>40);
Jiao et al. [182]	**Compound 38**	EGFR(0.54); HER2(54.37);	NCI-H358(0.618); PC-9(0.092); Calu-3(1.345); NCI-H1781(>2);
	Afatinib	EGFR(0.27); HER2(3.88);	NCI-H358(0.0086); PC-9(0.0009); Calu-3(0.0101); NCI-H1781(0.002);
	Poziotinib	EGFR(0.33); HER2(1.69);	NCI-H358(0.001); PC-9(0.00037); Calu-3(0.0027); NCI-H1781(0.00023);
Park et al. [28]	**Compound 39**	$EGFR^{wt}$(>50*10^4); $EGFR^{del19/T790M/C797S}$(17.9);	-
Zhou et al. [207]	**Compound 40**	$EGFR^{wt}$(>10^5); $EGFR^{L8585R/T790M}$(740); SI(>13.5)[i]	A549(14.33 ± 1.16); NCI-H460(17.81 ± 1.25); H1975(13.41 ± 1.14); Ba/F3-$EGFR^{Del19/T790M/C797S}$(91)[h]
	Afatinib	$EGFR^{wt}$(6); $EGFR^{L8585R/T790M}$(10);	-

		SI(0.6)[i]	
	AZD9291	EGFR^{wt}(28); EGFR$^{L8585R/T790M}$(12); SI(2.3)[i]	A549(0.66 ± 0.08); NCI-H460(-); H1975(0.073 ± 0.15); Ba/F3-EGFR$^{Del19/T790M/C797S}$(-)[h]
Dou et al. [211]	**Compound 41**	EGFR$^{L858R/T790M/C797S}$(128)	BaF3-EGFR$^{L858R/T790M/C797S}$ (0.75 ± 0.29); BaF3-EGFR$^{19del/T790M/C797S}$(0.09 ± 0.03);
	Vandetanib	EGFR$^{L858R/T790M/C797S}$(369)	BaF3-EGFR$^{L858R/T790M/C797S}$ (4.28 ± 0.17); BaF3-EGFR$^{19del/T790M/C797S}$(3.21 ± 0.49);
	Brigatinib	EGFR$^{L858R/T790M/C797S}$(8)	BaF3-EGFR$^{L858R/T790M/C797S}$ (0.56 ± 0.16); BaF3-EGFR$^{19del/T790M/C797S}$(0.17 ± 0.05);
Li et al. [206]	**Compound 42**	EGFR^{WT}(2.5 ± 0.8): EGFR$^{L858R/T790M}$(35.3 ± 12.1); EGFR$^{L858R/T790M/C797S}$(2.2 ± 1.1); EGFR$^{Del19/T790M/C797S}$(331.3±25.3);	BaF3 (7.68 ± 0.73); BaF3- EGFR$^{L858R/T790M/C797S}$(0.64 ± 0.14);H1975(3.03 ± 0.49); A431(1.24 ± 0.16);
	AZD9291	EGFR^{WT}(216.2±109.4): EGFR$^{L858R/T790M}$(2.8±2.0); EGFR$^{L858R/T790M/C797S}$(461.7±234.0);	BaF3 (5.11 ± 0.49); BaF3- EGFR$^{L858R/T790M/C797S}$(3.93 ± 0.38);H1975(0.03 ± 0.01); A431(1.44 ± 0.03);
	Brigatinib	EGFR^{WT}(35.9±2.8): EGFR$^{L858R/T790M}$(4.0±0.1); EGFR$^{L858R/T790M/C797S}$(1.6±0.1);	BaF3 (7.31 ± 0.98); BaF3- EGFR$^{L858R/T790M/C797S}$(0.42 ± 0.09);

[a] Growth inhibition [b]Selectivity Index (SI) = EGFRWT IC50 value/EGFRT790M IC50 value, [c] Expressed as inhibition ratios (%), [d] under hypoxia condition, [e] under hypoxia condition + irradiation; [f]SI = IC_{50} A549/ IC_{50} H1975; [g]SI = IC_{50} EGFR^{WT}: IC_{50} EGFR$^{L858R/T790M}$; [h] % Inhibition (10 μM); [I] SI = IC_{50} (EGFR^{WT})/IC_{50} (EGFR$^{L8585R/T790M}$); [j] % inhibition at 100nM
